# Unveiling Neonatal Pneumonia Microbiome by High-throughput Sequencing and Droplet Culturomics

**DOI:** 10.1093/gpbjnl/qzaf047

**Published:** 2025-05-29

**Authors:** Zerui Wang, Xin Cheng, Yibin Xu, Zhiyi Wang, Liyan Ma, Caiming Li, Shize Jiang, Yuchen Li, Shuilong Guo, Wenbin Du

**Affiliations:** Biomedical Sciences College & Shandong Medical Biotechnology Center, Shandong First Medical University & Shandong Academy of Medical Sciences, Jinan 250117, China; State Key Laboratory of Microbial Diversity and Innovative Utilization, Institute of Microbiology, Chinese Academy of Sciences, Beijing 100101, China; Clinical Laboratory Center, Beijing Friendship Hospital, Capital Medical University, Beijing 100050, China; State Key Laboratory of Microbial Diversity and Innovative Utilization, Institute of Microbiology, Chinese Academy of Sciences, Beijing 100101, China; State Key Laboratory of Microbial Diversity and Innovative Utilization, Institute of Microbiology, Chinese Academy of Sciences, Beijing 100101, China; Clinical Laboratory Center, Beijing Friendship Hospital, Capital Medical University, Beijing 100050, China; State Key Laboratory of Microbial Diversity and Innovative Utilization, Institute of Microbiology, Chinese Academy of Sciences, Beijing 100101, China; State Key Laboratory of Microbial Diversity and Innovative Utilization, Institute of Microbiology, Chinese Academy of Sciences, Beijing 100101, China; Biomedical Sciences College & Shandong Medical Biotechnology Center, Shandong First Medical University & Shandong Academy of Medical Sciences, Jinan 250117, China; State Key Laboratory of Microbial Diversity and Innovative Utilization, Institute of Microbiology, Chinese Academy of Sciences, Beijing 100101, China; Department of Gastroenterology, Beijing Friendship Hospital, Capital Medical University, Beijing 100050, China; Biomedical Sciences College & Shandong Medical Biotechnology Center, Shandong First Medical University & Shandong Academy of Medical Sciences, Jinan 250117, China; State Key Laboratory of Microbial Diversity and Innovative Utilization, Institute of Microbiology, Chinese Academy of Sciences, Beijing 100101, China; Medical School and College of Life Sciences, University of the Chinese Academy of Sciences, Beijing 100049, China

**Keywords:** Neonatal pneumonia, Respiratory microbiota, Droplet microfluidics, Single-cell cultivation, Host–pathogen interaction

## Abstract

Neonatal pneumonia is a leading cause of infant mortality worldwide; however, a lack of microbial profiling, especially of low-abundance species, makes accurate diagnosis challenging. Traditional methods can fail to capture the complexity of the neonatal respiratory microbiota, thereby obscuring its role in disease progression. Here, we describe a novel approach that combines high-throughput sequencing with droplet-based microfluidic cultivation to investigate microbiome shifts in neonates with pneumonia. Using 16S ribosomal RNA (rRNA) gene sequencing of 71 pneumonia cases and 49 controls, we identified 1009 genera, including 930 low-abundance taxa, which showed significant compositional differences between groups. Linear discriminant analysis effect size identified key pneumonia-associated genera, such as *Streptococcus*, *Rothia*, and *Corynebacterium*. Droplet-based cultivation recovered 299 strains from 94 taxa, including rare species and ESKAPE pathogens, thereby supporting targeted antimicrobial management. Host–pathogen interaction assays showed that *Rothia* and *Corynebacterium* induced inflammation in lung epithelial cells, likely via dysregulation of the PI3K-Akt pathway. Integrating these marker taxa with clinical factors, such as gestational age and delivery type, offers the potential for precise diagnosis and treatment. The recovery of diverse species can support the construction of a biobank of neonatal respiratory microbiota to advance mechanistic studies and therapeutic strategies.

## Graphical abstract

**Graphical Abstract:**
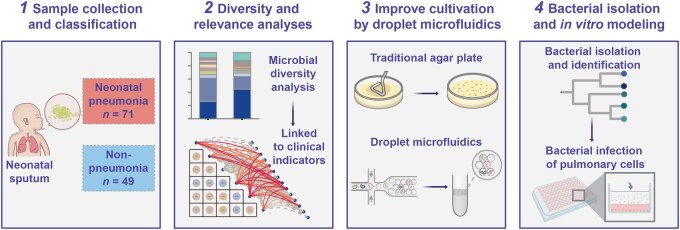

## Introduction

Neonatal pneumonia is a critical global health challenge, causing between 750,000 and 1.2 million infant deaths annually and ranking as one of the leading causes of neonatal morbidity and mortality [1]. Despite advances in neonatal care, the early diagnosis and management of neonatal pneumonia remain challenging due to limitations in traditional diagnostic methods, which often fail to detect low-abundance or difficult-to-culture pathogens [2]. These shortcomings delay targeted interventions, thereby increasing the risk of poor patient outcomes. Thus, innovative approaches are urgently needed to enhance pathogen identification and improve care for neonates with pneumonia.

Neonatal health is strongly regulated by the neonate’s respiratory microbiota, which forms a protective barrier against pathogens through competition and immune modulation [3–5]. Consequently, disruptions to the microbiota balance increase the neonate’s susceptibility to respiratory infections, leading to significant short-term and long-term health issues [6–8]. The neonatal microbiome is influenced by multiple factors, including the neonate’s mode of delivery (*i.e.*, vaginal birth or cesarean section) [9], gestational age [8], breastfeeding [10], and early antibiotic exposure [11]. However, microbial profiling of specimens collected from neonates is complicated by small sample volumes and low microbial density. One approach that offers significant promise for identifying microbial shifts associated with neonatal pneumonia is next-generation sequencing (NGS), as it enables the detailed profiling of microbial diversity [12,13]. However, sequencing alone cannot fully address key clinical challenges, such as isolating specific pathogens or understanding their pathogenic mechanisms. In neonates, these issues are compounded by the presence of low-abundance or commensal species, which may become opportunistic pathogens but can often remain undetected using traditional cultivation methods that are biased toward fast-growing bacteria [14]. Thus, the analysis of the neonatal microbiota requires innovative technologies, such as droplet-based microfluidic single-cell cultivation [15–17], as they compartmentalize microbes, providing single cells with equal space and an environment conducive to growth without competition. However, these new tools have not yet been applied to the cultivation and characterization of the neonatal respiratory microbiota.

In this study, our aim was to address the clinical and methodological gaps in neonatal microbial analysis by investigating the composition of the respiratory microbiota and its potential pathogenic mechanisms in neonatal pneumonia. We integrated high-throughput sequencing with droplet-based microfluidic single-cell cultivation to enhance the recovery of low-abundance microorganisms from infected neonatal lungs. We also explored the pathogenic effects of microbiota species using transcriptomic sequencing of lung epithelial cells co-cultured with pneumonia-enriched bacterial strains. This combined approach provides a deeper understanding of microbial shifts occurring in neonatal pneumonia and highlights the benefits of novel cultivation methods for advancing diagnostic accuracy and therapeutic interventions in neonates with pneumonia.

## Results

### Overview of the study design and clinical cohort

**Figure 1:**
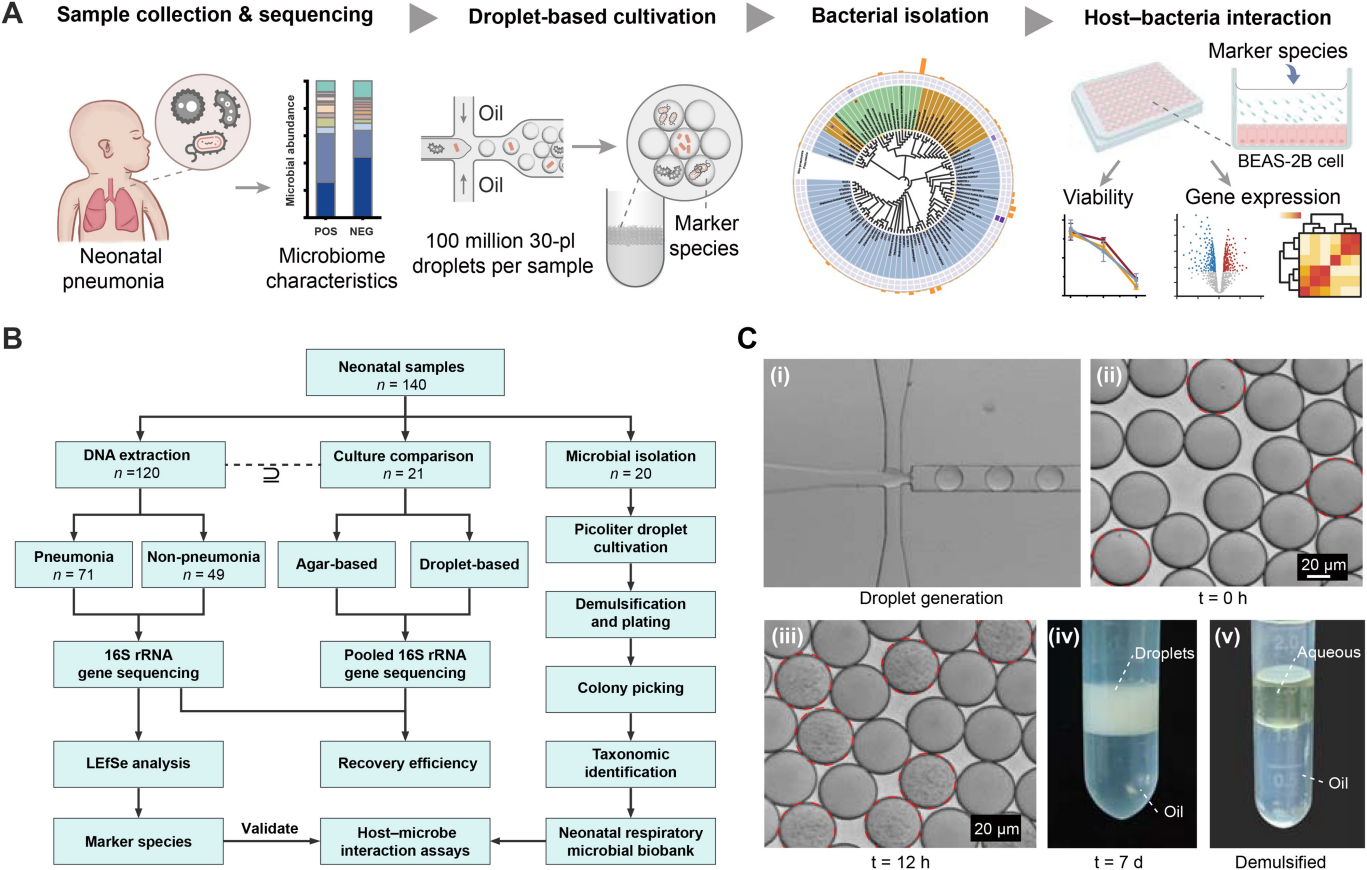
Study design and workflow for characterizing the neonatal respiratory microbiome **A**. Schematic representation of the study. High-throughput sequencing and droplet-based microfluidic cultivation were used to analyze microbial diversity, recover cultivable species, and perform host–bacteria interaction studies to identify potential pathogenic taxa. POS and NEG represent the neonatal pneumonia and non-pneumonia groups, respectively. **B**. Overview of the sample groups, experimental workflows, and methodologies applied. **C**. Droplet-based cultivation process: (i) flow-focusing droplet generation, (ii–iv) incubation of droplets, and (v) demulsification for recovery and plating of neonatal respiratory microbiota. Scale bar, 20 μm. rRNA, ribosomal RNA; LEfSe, linear discriminant analysis effect size; t, time; d, day.

The limited microbial load and small sample volumes in neonatal sputum pose challenges for early diagnosis and antimicrobial management of neonatal pneumonia. To overcome these limitations, this study employed a combination of high-throughput sequencing and advanced droplet-based microfluidic single-cell cultivation to identify microbial communities and potential pathogenic species associated with neonatal pneumonia (**Figure 1**A). In 2023, a total of 140 sputum samples were collected from neonates, including 71 pneumonia cases and 49 non-pneumonia controls, to analyze microbial diversity and abundance (Figure 1B; **Table 1**). Droplet-based microfluidic cultivation was applied to 21 samples to enable a direct comparison with traditional agar plate cultivation. A further 20 samples underwent exclusive droplet-based bacterial isolation to evaluate the efficiency of droplet-based analysis for detecting and isolating low-abundance taxa associated with neonatal pneumonia (Figure 1C). *In vitro* host–pathogen interaction analyses were also performed to assess the pathogenic potential of key bacteria enriched in pneumonia samples to obtain mechanistic insights into the microbial contributions to the pathogenesis of neonatal pneumonia.

**Table 1:** Clinical and demographic characteristics of neonates with or without pneumonia included in the study cohort

Variable	Classification	Neonatal pneumonia (*n* = 71)	Non-pneumonia (*n* = 49)	t/χ^2^	*P*
Gender	Male	57.7% (41/71)	55.1% (27/49)	0.083^a^	0.774
	Female	42.3% (30/71)	44.9% (22/49)		
Age (day)	Day 0	31.0% (22/71)	59.2% (29/49)	10.047^a^	0.034*^c^
	Days 1–7	29.6% (21/71)	16.3% (8/49)		
	Days 8–14	5.6% (4/71)	4.1% (2/49)		
	Days 15–21	5.6% (4/71)	6.1% (3/49)		
	Days 22–28	28.2% (20/71)	14.3% (7/49)		
Mode of delivery	Caesarean section	54.9% (39/71)	59.2% (29/49)	0.214^a^	0.644
	Vaginal delivery	45.1% (32/71)	40.8% (20/49)		
Gestational age	Premature birth	36.6% (26/71)	44.9% (22/49)	0.828^a^	0.363
	Full-term	63.4% (45/71)	55.1% (27/49)		
Hematological indicator (mean ± SD)	WBC (10^9^/l)	12.86 ± 4.82	12.92 ± 4.86	−0.063^b^	0.95
	Neu (10^9^/l)	6.19 ± 4.63	7.38 ± 4.70	−1.378^b^	0.171
	Lym (10^9^/l)	4.42 ± 2.21	4.23 ± 1.41	0.527^b^	0.599
	NLR	1.58 ± 1.31	2.23 ± 2.88	−1.631^b^	0.106
	RBC (10^12^/l)	3.95 ± 0.82	4.28 ± 0.75	−2.249^b^	0.026*
	HGB (g/l)	132.02 ± 33.59	151.78 ± 31.09	−3.219^b^	0.002**

*Note*:  ^a^, Chi-square test. The *P* values are based on the two-tailed Fisher’s exact test (used when < 5 observations in each group) and χ^2^ tests (used when > 5 observations in each group). ^b^, independent ^t^-test. ^c^, the intergroup differences occurred between Day 0 and Days 1–7 (*P* = 0.019), as well as between Day 0 and Days 22–28 (*P* = 0.016). *, *P* < 0.05; **, *P* < 0.01. SD, standard deviation; WBC, white blood cell; Neu, neutrophil; Lym, lymphocyte; NLR, neutrophil-to-lymphocyte ratio; RBC, red blood cell; HGB, hemoglobin.

No significant differences were found between the pneumonia and non-pneumonia groups in terms of gender, mode of birth delivery, gestational age, or key hematological markers, such as white blood cell (WBC) count, neutrophil (Neu) count, lymphocyte (Lym) count, and neutrophil-to-lymphocyte ratio (NLR). However, the postnatal age distribution showed a significant difference (*P* = 0.034), with notable variations between Day 0 and Days 1–7 (*P* = 0.019) and between Day 0 and Days 22–28 (*P* = 0.016). The pneumonia group also had significantly higher levels of red blood cells (RBCs; *P* = 0.026) and hemoglobin (HGB; *P* = 0.002) compared to the non-pneumonia controls.

### Altered respiratory microbiota of neonates with pneumonia

**Figure 2:**
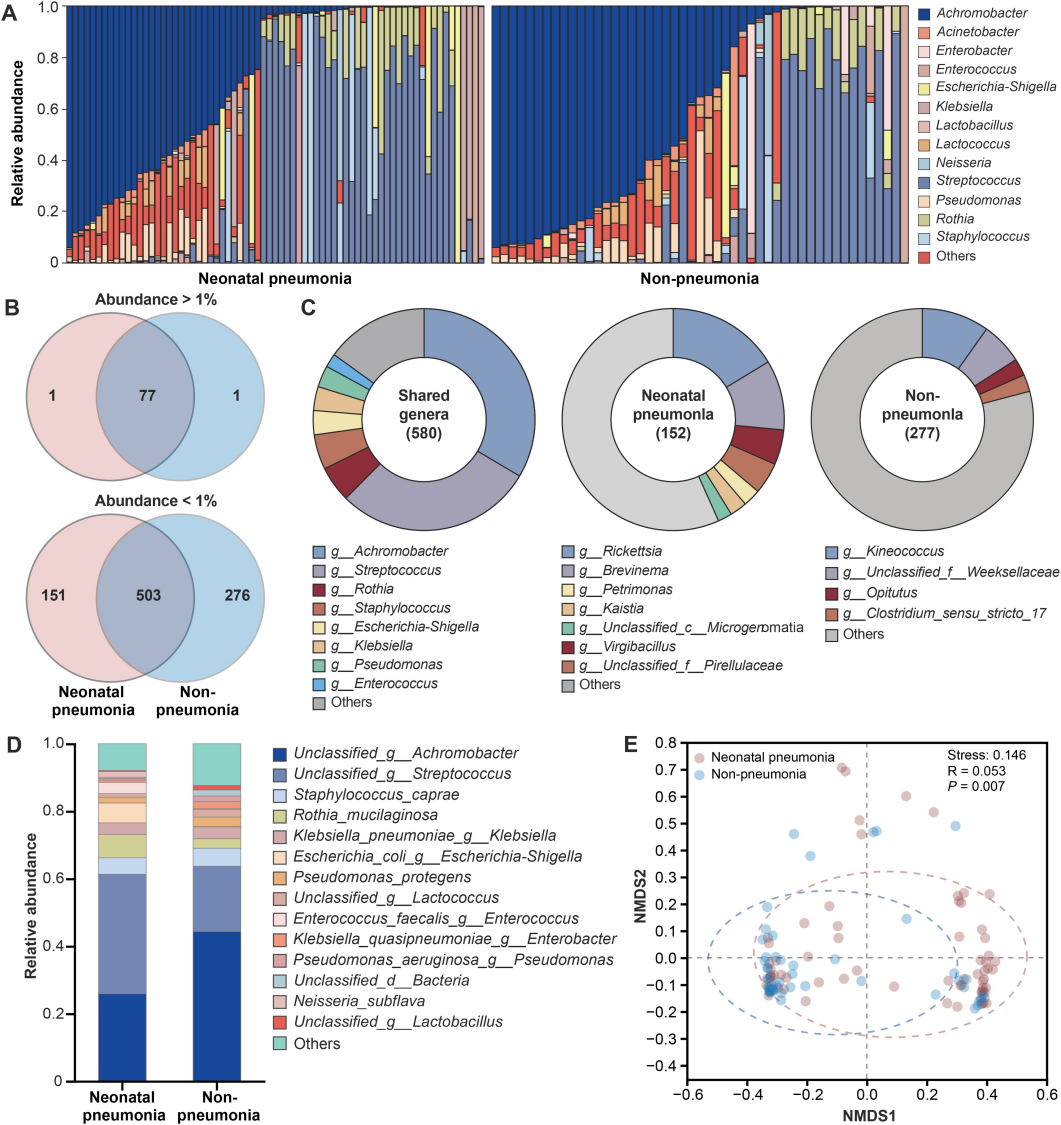
Comparison of microbial composition and diversity in neonatal pneumonia and non-pneumonia groups **A**. Bacterial composition at the genus level, with taxa below 1% relative abundance grouped as “Others”. **B**. Venn diagrams showing the overlap of high-abundance and low-abundance genera between the neonatal pneumonia and non-pneumonia groups. **C**. Pie charts illustrating shared and unique genera in the two groups, with taxa below 1% relative abundance grouped as “Others”. **D**. Bacterial composition at the species level. **E**. β-diversity analysis using NMDS to compare microbial communities between two groups, with stress = 0.146, R = 0.053, and *P* = 0.007. NMDS, non-metric multidimensional scaling.

The respiratory microbiota of neonates with pneumonia was compared to that of neonates without pneumonia at both the genus and species levels using raw sequencing data (**Figure 2**). The pneumonia group exhibited elevated levels of *Streptococcus*, *Rothia*, *Escherichia*-*Shigella*, *Neisseria*, and *Enterococcus* but lower levels of *Achromobacter*, *Pseudomonas*, *Lactococcus*, *Lactobacillus*, and unclassified bacterial taxa (Figure 2A). Depiction of the overlap of high-abundance and low-abundance genera between two groups as Venn diagrams (Figure 2B) and the unique and shared genera as pie charts (Figure 2C) identified *Achromobacter*, *Streptococcus*, *Rothia*, *Staphylococcus*, *Escherichia*-*Shigella*, *Klebsiella*, *Pseudomonas*, and *Enterococcus* as shared genera. The pneumonia cases contained unique genera, including *Rickettsia*, *Brevinema*, and *Virgibacillus*, while the non-pneumonia cases included unique genera, such as *Kineococcus*, *Opitutus*, and *Clostridium_sensu_stricto_17*. Further differences at the species level between the pneumonia and non-pneumonia groups are shown in Figure 2D.

Analysis of the microbial differences using α-diversity and β-diversity analyses revealed no significant differences (*P* > 0.05) (Figure S1A–D) in the α-diversity indices, including Shannon, observed species (Sobs), Simpson, and Coverage. However, the β-diversity indices, analyzed using non-metric multidimensional scaling (NMDS), revealed a clear separation between the two groups after dimensionality reduction (Figure 2E). Consistently, the analysis of similarities confirmed significantly greater distances between the groups than within the groups (*P =* 0.006) (Figure S1E). These findings suggest that while the overall microbial diversity remained consistent, differences in community composition and the relative abundance of specific taxa could contribute to the pathogenesis of neonatal pneumonia.

### Comparative analysis of marker microbial taxa

**Figure 3:**
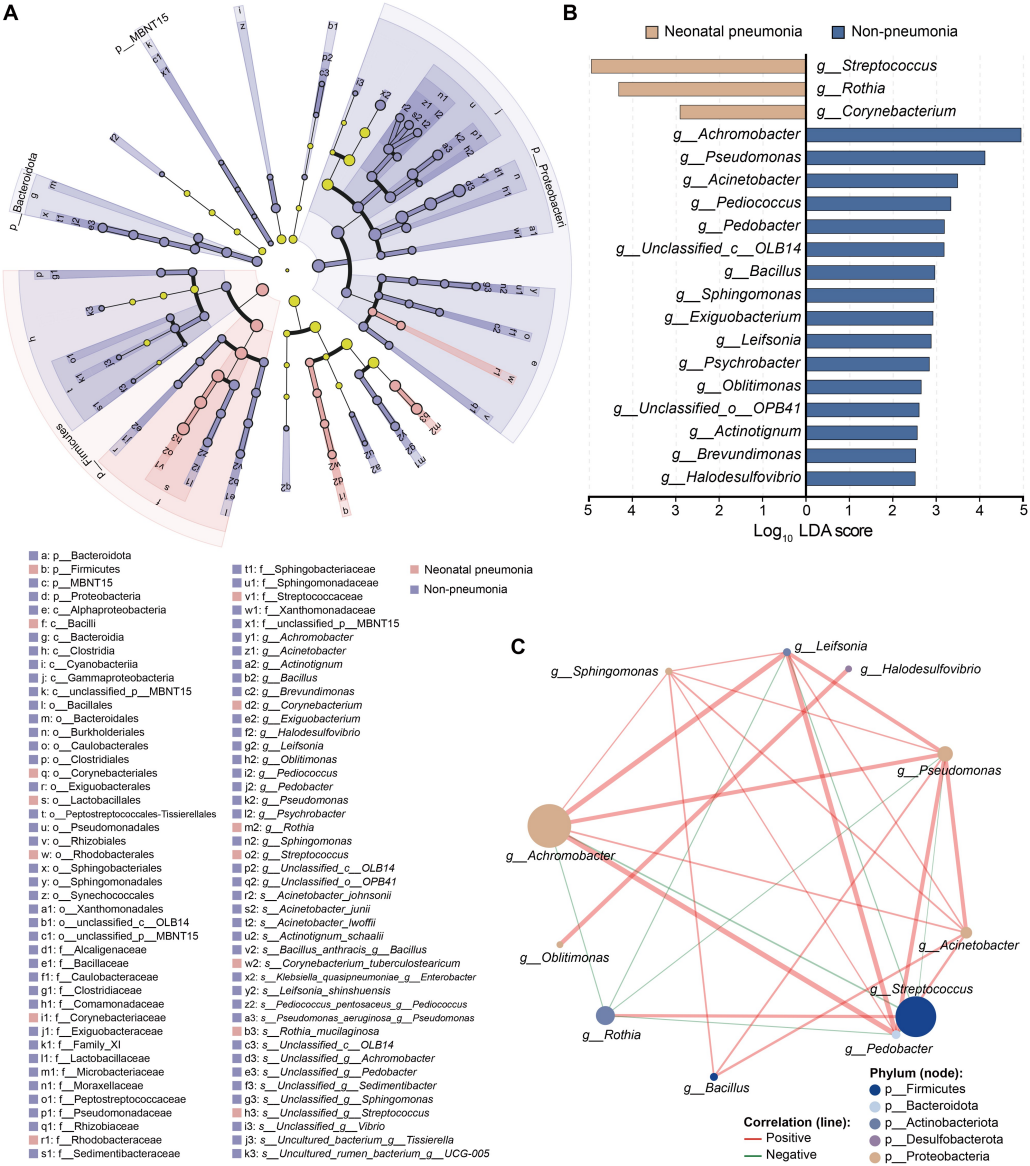
Core bacterial taxa and microbial interactions in neonatal pneumonia **A**. LEfSe cladogram displaying differentially abundant taxa between neonatal pneumonia and non-pneumonia groups from the phylum to species level (*P* < 0.05). Pink nodes indicate taxa enriched in the neonatal pneumonia group, purple nodes indicate taxa enriched in the non-pneumonia group, and yellow nodes represent non-differential taxa. Node size corresponds to the relative abundance. **B**. Key biomarkers (log_10_ LDA score > 2.5) for pneumonia (light brown) and non-pneumonia (blue) at the genus level. **C**. Network analysis at the genus level reveals significant microbial interactions in the neonatal pneumonia group. Line color represents positive (red) or negative (green) correlations, and line thickness corresponds to the strength of the correlation between variables. Node size reflects genus abundance, and node color represents phyla. LDA, linear discriminant analysis.

The raw sequencing data were subjected to linear discriminant analysis effect size (LEfSe) to identify significant microbial biomarkers across taxonomic levels, from phylum to species, that could differentiate neonatal pneumonia cases from non-pneumonia cases. The key genera enriched in the pneumonia group included *Streptococcus*,* Rothia*, and *Corynebacterium*, while the non-pneumonia group showed enrichment in *Achromobacter*, *Acinetobacter*, *Pediococcus*, *Pedobacter*, *Bacillus*, *Pseudomonas*, *Sphingomonas*, *Leifsonia*, *Exiguobacterium*, and others (**Figure 3**A). Further comparative abundance analyses of the dominant bacterial taxa are provided in Figure S2. Linear discriminant analysis (LDA) revealed a broader range of biomarkers in the non-pneumonia group than in the pneumonia group, indicating greater microbial heterogeneity in healthy neonates (Figure 3B).

The network analysis using Sparse Correlations for Compositional Data (SparCC) revealed key interactions within the microbial communities of both groups (Figure 3C, Figure S3). Highly abundant genera, such as *Achromobacter*, *Pseudomonas*, and *Streptococcus*, exhibited numerous correlations, suggesting their central role in structuring microbial networks. For example, *Achromobacter* and *Pseudomonas* displayed strong positive correlations with multiple taxa, indicating synergistic interactions. In contrast, *Streptococcus*, which is a key pneumonia-associated genus, showed both positive and negative correlations, suggesting its participation in both cooperative and competitive interactions within the microbial ecosystem. Overall, 34 nodes were identified in the pneumonia network and 39 in the non-pneumonia network, highlighting the great complexity and diversity of microbial interactions in the neonatal respiratory microbial communities (Figure S3). Furthermore, functional predictions of microbial communities using Phylogenetic Investigation of Communities by Reconstruction of Unobserved States 2 (PICRUSt2) revealed significant metabolic differences between the groups. Notably, the pneumonia group exhibited increased activity in pathways related to purine metabolism, amino acid biosynthesis, and peptidoglycan maturation, suggesting metabolic adaptations linked to microbial community changes and disease progression (Figure S4; File S1).

### Correlation analysis between driving factors, clinical indicators, and bacterial taxa

**Figure 4:**
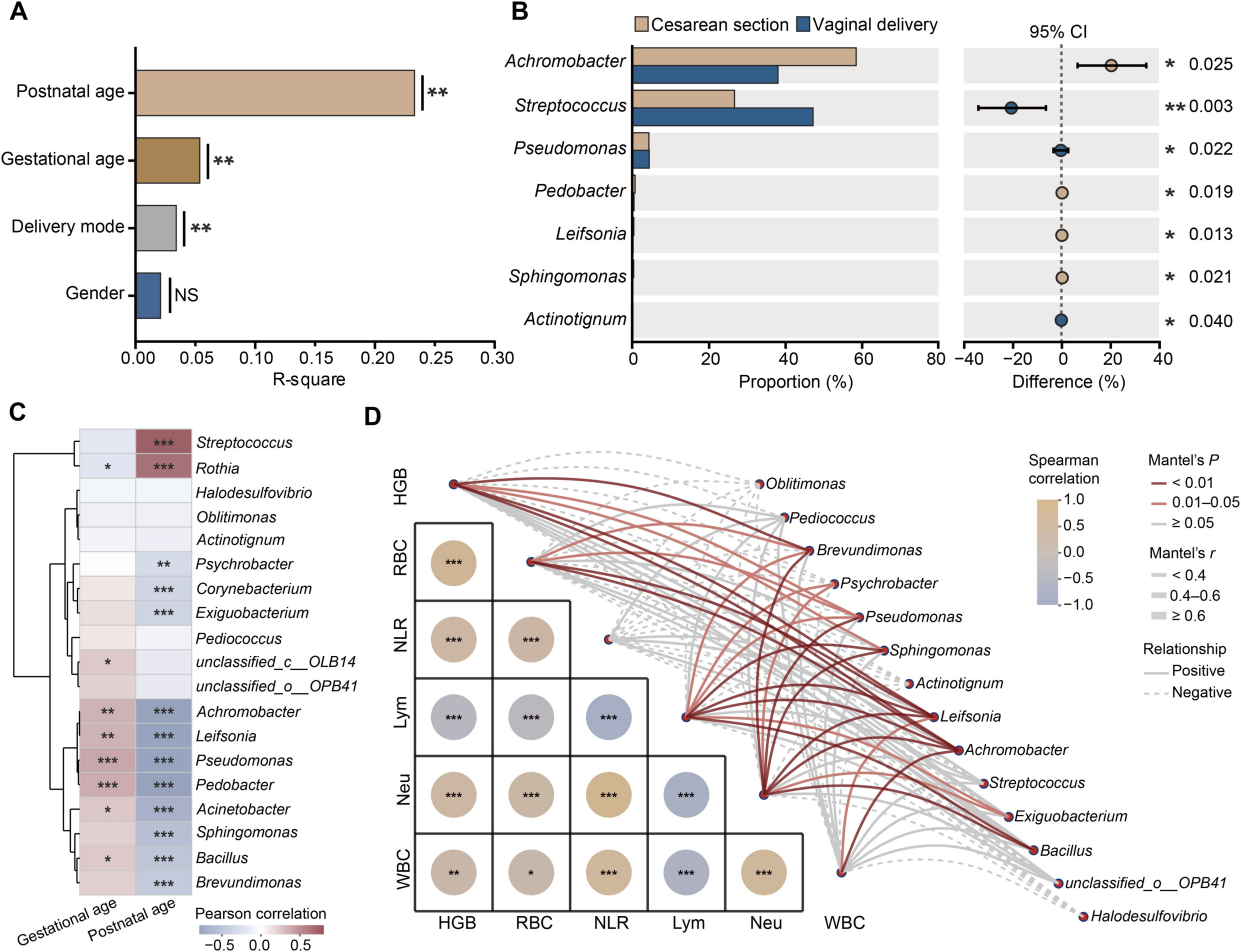
Correlation analysis between microbial composition, environmental factors, and clinical indicators **A**. PERMANOVA analysis showing the effect of environmental factors on microbial composition at the genus level. **, *P* < 0.01; NS, not significant (Bray-Curtis distance). **B**. Extended error bar plot of genera significantly differing between cesarean and vaginal delivery groups (Mann–Whitney U test). **C**. Heatmap showing correlations between bacterial taxa significantly enriched in the pneumonia or non-pneumonia group and gestational or postnatal age (Spearman correlation). **D**. Partial Mantel test results showing correlations between clinical indicators and bacterial communities significantly enriched in the pneumonia or non-pneumonia group. Line thickness indicates the Mantel’s *r* statistic (categorized as < 0.4, 0.4–0.6, or ≥ 0.6), line color represents the Mantel’s *P* statistic, and line style denotes positive (solid) or negative (dashed) correlations. Heatmap is color-coded based on Spearman correlation coefficients (*, *P* < 0.05; **, *P* < 0.01; ***, *P* < 0.001). PERMANOVA, permutational multivariate analysis of variance; CI, confidence interval; HGB, hemoglobin; RBC, red blood cell; NLR, neutrophil-to-lymphocyte ratio; Lym, lymphocyte; Neu, neutrophil; WBC, white blood cell.

Permutational multivariate analysis of variance (PERMANOVA) analysis using Bray-Curtis distances was performed on the raw sequencing data to better understand the factors influencing microbial composition differences between neonates with and without pneumonia. This analysis identified postnatal age, gestational age, and mode of delivery as significant contributors to microbial composition variability, whereas gender had no significant effect (**Figure 4**A). Specifically, *Achromobacter*, *Pedobacter*, *Leifsonia*, and *Sphingomonas* were more prevalent in neonates delivered by cesarean section, while *Streptococcus*, *Pseudomonas*, and *Actinotignum* were more abundant in neonates delivered vaginally (Figure 4B).

Correlation analysis further revealed that the bacterial taxa significantly enriched in the neonatal pneumonia or non-pneumonia group showed varying degrees of association with gestational and postnatal age (Figure 4C). For example, *Achromobacter*, *Pseudomonas*, *Pedobacter*, *Leifsonia*, *Acinetobacter*, and *Bacillus* were positively correlated with gestational age, while *Rothia* showed a negative correlation. Postnatal age was positively correlated with the presence of *Streptococcus* and *Rothia*, while genera such as *Psychrobacter*, *Corynebacterium*, *Exiguobacterium*, *Achromobacter*, *Leifsonia*, *Pseudomonas*, *Pedobacter, Acinetobacter*, *Sphingomonas*, *Bacillus*, and *Brevundimonas* were negatively associated with postnatal age. The use of the Mantel test to explore microbial relationships with clinical diagnostic indicators revealed significant correlations between dominant bacterial taxa and clinical parameters (Figure 4D). For example, *Leifsonia* was positively associated with HGB, RBC, NLR, Lym, Neu, and WBC; *Sphingomonas* showed positive correlations with HGB, Lym, and Neu; *Achromobacter* was positively associated with HGB, RBC, Lym, Neu, and WBC; and *Brevundimonas* was positively associated with HGB, RBC, Lym, and Neu. These findings suggest that specific bacterial taxa may be linked to clinical indicators relevant to neonatal health and disease progression.

### Use of droplet-based microfluidic technology to improve the cultivability of microorganisms

**Figure 5:**
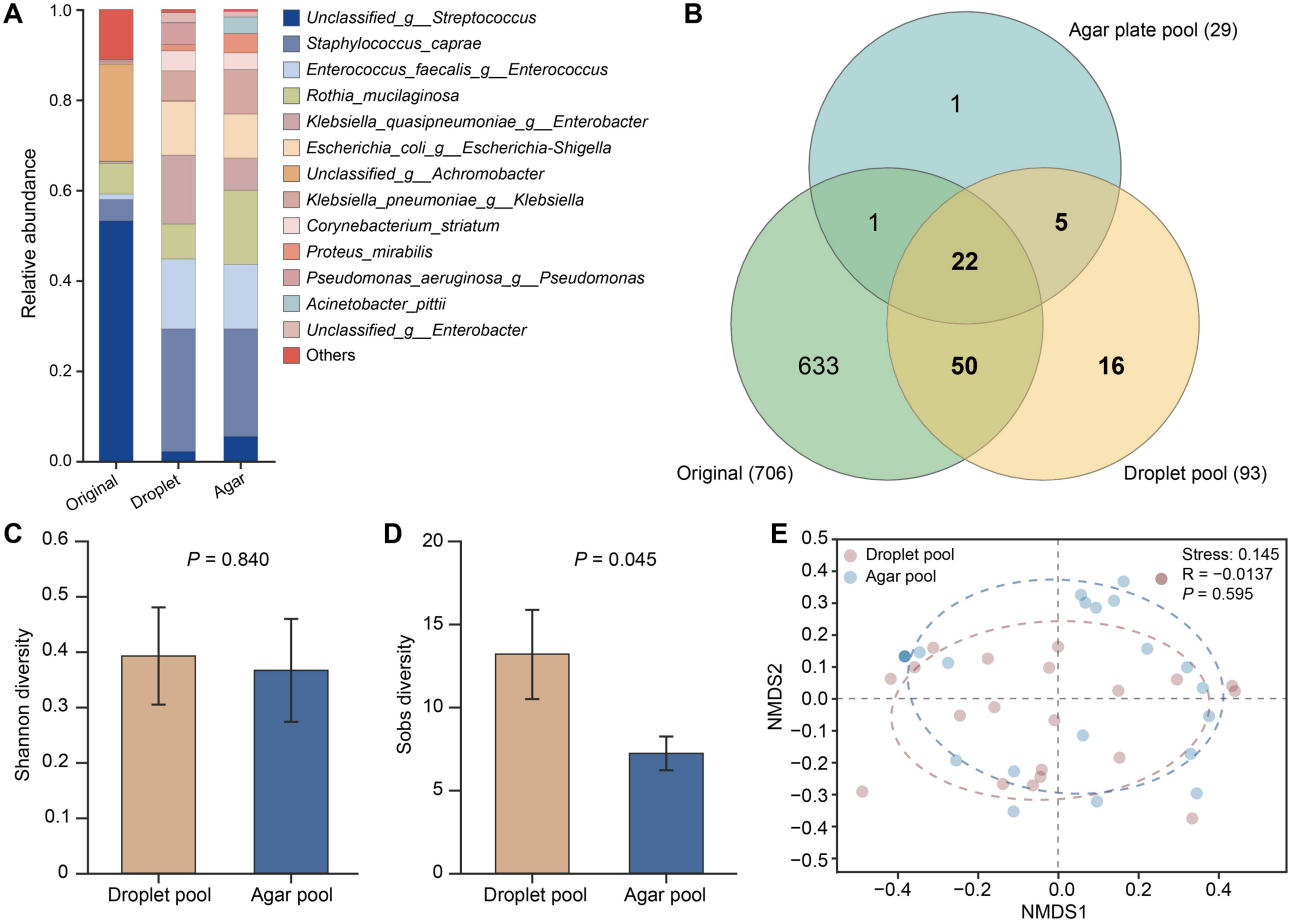
Droplet-based microfluidic cultivation significantly improved microbial recovery compared to traditional agar plate cultivation **A**. Species-level microbial composition, with species of average abundance > 0.01 shown and others grouped as “others”. **B**. Venn diagram illustrating shared and unique bacterial compositions among original samples, droplet-based cultivation (droplet pool), and agar-based cultivation (agar pool). **C**. and** D**. α*-*diversity comparisons using Shannon diversity (C) and Sobs diversity (D). **E**. β-diversity analysis via NMDS comparing microbial community structures between droplet-based and agar-based methods. Sobs, observed species.

The limited sample volume and low microbial abundance in neonatal respiratory sputum samples pose significant challenges to the accurate identification of the causative pathogens and the mechanisms underlying neonatal pneumonia. This is because traditional cultivation, which remains the gold standard for diagnosing bacterial infections, often has low detection rates and requires lengthy cultivation periods [13]. We overcame these limitations by developing a high-throughput droplet-based microfluidic platform to enhance microbial recovery. We tested the effectiveness of our platform by comparing the microbial community composition at the species level across the original samples and samples after droplet-based cultivation and agar plate cultivation (**Figure 5**A). Bacteria with relative abundances below 1% were grouped as “others”. The resulting Venn diagram highlighted a greater species overlap between the original samples and the droplet pool (72 species) compared to the agar pool (23 species), demonstrating that the droplet-based method recovered over three times the number of species achieved using agar plates (Figure 5B).

The Shannon diversity indices showed no significant differences between the droplet-based and agar-based methods (*P* = 0.840) (Figure 5C). However, the Sobs index showed the recovery of a significantly higher number of species using droplet-based cultivation than using agar plate cultivation (*P* = 0.045) (Figure 5D). Similarly, β-diversity analysis revealed no distinct clustering between the two methods (*P* = 0.595) (Figure 5E). Overall, the droplet-based method was better at capturing rare and low-abundance species, which is in agreement with our previous studies [16].

The droplet generation capacity was also significantly increased from our previous 1 × 10^4^ nl droplets to 1 × 10^8^ pl droplets, enabling more effective recovery of low-abundance species. Despite their significant diversity in the host microbiome, these low-abundance bacteria, often representing less than 1% of the relative abundance, are frequently overlooked in microbiome studies. Our high-throughput sequencing of neonatal sputum samples confirmed that low-abundance species outnumbered high-abundance taxa in terms of diversity (Figure 2). Importantly, the droplet-based cultivation enriched 60% (12/20) of the marker species identified through LEfSe analysis (Table S1), thereby outperforming traditional agar cultivation, which recovered only 20% (4/20).

### Microbial diversity and phylogeny from droplet-based cultivation

**Figure 6:**
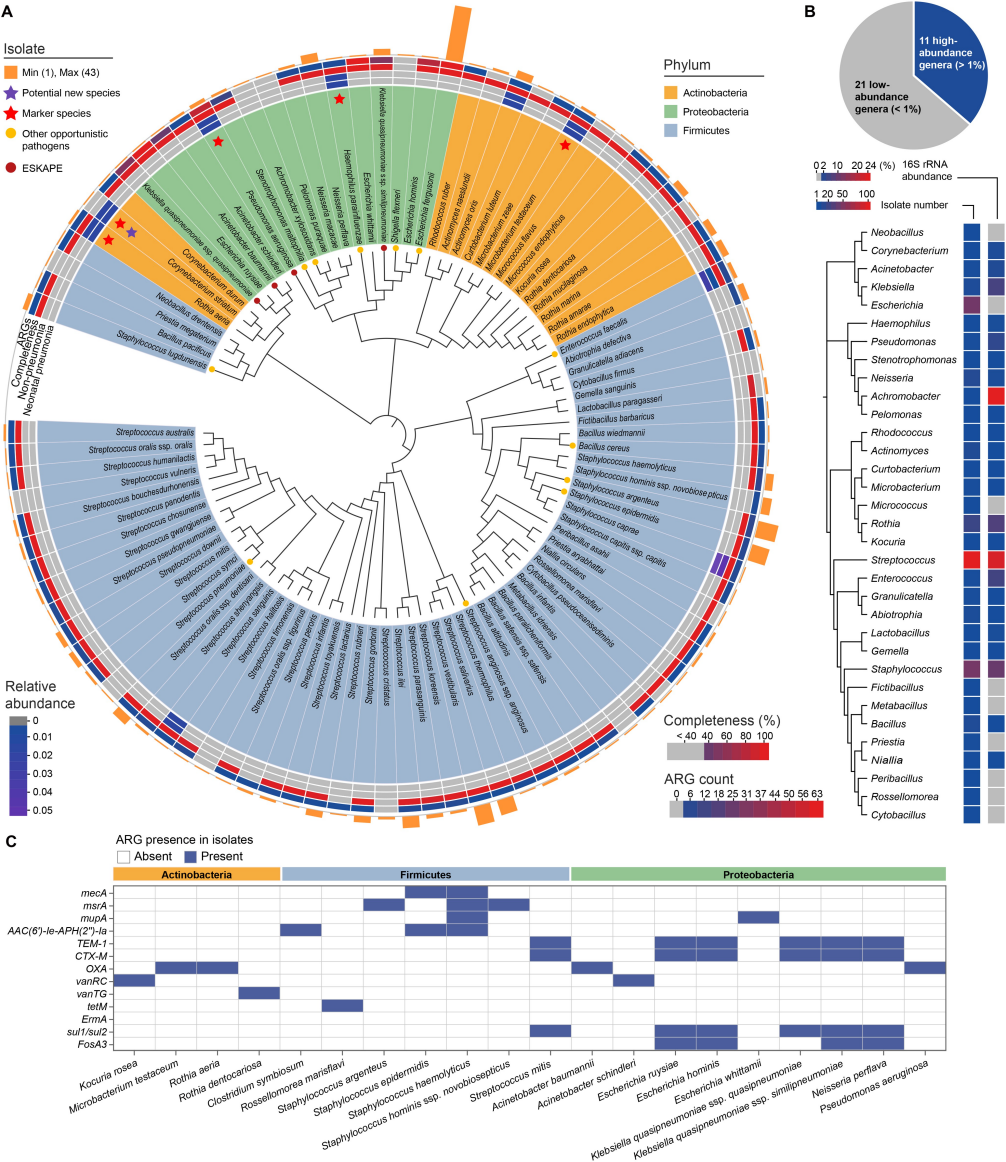
Phylogenetic diversity and ARG of bacterial strains isolated via droplet-based cultivation **A**. Phylogenetic tree at the species level showing most isolates classified under Firmicutes (blue), Actinobacteria (orange), and Proteobacteria (green). Symbols indicate specific taxa of interest: purple star — potential novel species (< 98.47% similarity); rose red star — marker species; brown dot — ESKAPE pathogens; and yellow dot — other opportunistic pathogens. Circular heatmap illustrates (from the innermost to outermost rings): the relative abundance of bacterial isolates from the neonatal pneumonia group; the relative abundance of bacterial isolates from the non-pneumonia group; the completeness of each isolate’s whole-genome assembly; the highest number of ARGs identified in its isolates; and the number of unique strains isolated. **B**. Phylogenetic tree at the genus level showing the number of isolates and their relative abundance based on high-throughput sequencing. The accompanying pie chart displays the proportion of high-abundance (> 1%) and low-abundance (< 1%) species within the samples. **C**. Whole-genome sequencing revealed the distribution of ARGs with major implications for neonatal health across the isolates, including those associated with MRSA and staphylococcal resistance, vancomycin resistance, ESBLs and β-lactamases in gram-negative bacteria, and sulfonamide resistance. ARG, antimicrobial resistance gene; MRSA, methicillin-resistant *Staphylococcus aureus*; ESBL, extended-spectrum β-lactamase.

Our use of droplet-based single-cell cultivation enabled the isolation of 299 bacterial strains from 20 neonatal sputum samples. Among the recovered strains, there were 95 unique species spanning three major phyla: Firmicutes (65.3%), Actinobacteria (17.9%), and Proteobacteria (16.8%) (**Figure 6**A; Table S2). A phylogenetic tree constructed from full-length 16S ribosomal RNA (rRNA) gene sequences highlighted the broad taxonomic diversity of the isolates, with Firmicutes emerging as the most abundant phylum, consistent with the amplicon sequencing results from the original sputum samples.

Both pathogenic and commensal marker species were successfully recovered using droplet-based cultivation. In total, 16 opportunistic pathogenic species were identified, including ESKAPE pathogens such as *Pseudomonas aeruginosa*, *Acinetobacter baumannii*, and *Klebsiella quasipneumoniae*, emphasizing the importance of recovering these organisms in neonatal pneumonia cases for antimicrobial susceptibility testing. Notably, one strain exhibited 98.47% 16S rRNA gene similarity to *Corynebacterium striatum*, suggesting this as a novel pneumonia-related species. While *C. striatum* is typically a harmless commensal bacterium in the nasopharynx, it has been increasingly recognized as an opportunistic pathogen in hospital settings, underscoring its potential clinical relevance [18].

The droplet-based platform also demonstrated superior sensitivity in terms of cultivating the low-abundance species identified through the high-throughput sequencing of neonatal sputum samples. These rare taxa, often missed by traditional cultivation methods, are highlighted in the heatmaps alongside the phylogenetic trees (Figure 6B). This capability for the cultivation of these species provides a more comprehensive view of the neonatal respiratory microbiome and its potential role in disease pathogenesis.

Further characterization of these low-abundance isolates by whole-genome sequencing (WGS) of 167 representative strains resulted in the identification of 80 distinct species with high genome completeness (Table S3). The highest number of antibiotic resistance genes (ARGs) annotated in these genomes is displayed in the fourth ring (from the innermost to the outermost) of the phylogenetic tree (Figure 6A). The key ARGs relevant to neonatal infections included genes conferring methicillin resistance (*e.g.*, *mecA* and *mupA*), vancomycin resistance (*e.g.*, *vanRC* and *vanTG*), and β-lactamase [including extended-spectrum β-lactamases (ESBLs); *e.g.*, *TEM-1* and *CTX-M*] (Figure 6C). The ability to isolate marker species with established or emerging pathogenicity, coupled with high-resolution ARG profiling, highlights the diagnostic potential of droplet-based cultivation.

### Host–pathogen interaction assays reveal potential mechanisms of epithelial cell injury

**Figure 7:**
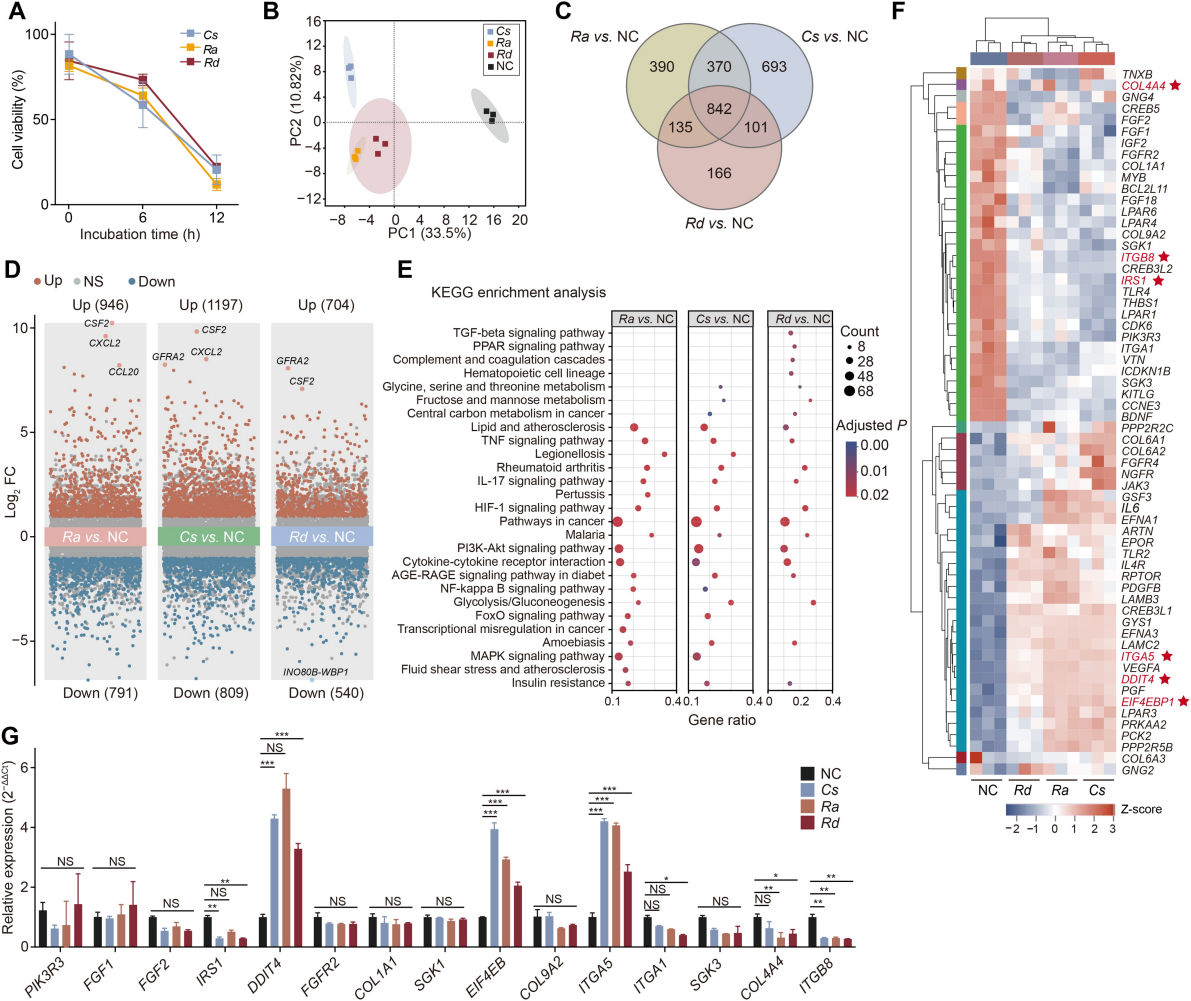
Host–pathogen interaction assays reveal the inflammatory effects of ***Cs***, ***Ra***, and ***Rd*** on pulmonary cells (BEAS-2B) **A**. Normalized cell viability of BEAS-2B cells treated with *Cs*, *Ra*, or *Rd*, assessed using the CCK-8 assay and compared to the NC group. Data are presented as mean ± SD from three independent experiments, showing significant inhibition of cell proliferation. **B**. PCA of transcriptomic profiles shows clear clustering of bacterial treatment groups and NC, indicating distinct gene expression patterns. **C**. Venn diagram displaying the overlap and uniqueness of DEGs among the *Cs*, *Ra*, and *Rd* treatment groups compared to NC. **D**. Volcano plots illustrating the upregulated (red) and downregulated (blue) DEGs for each bacterial treatment group *vs.* NC. **E**. KEGG enrichment analysis of DEGs identifies the top 20 significantly enriched pathways, including the PI3K-Akt signaling pathway, cytokine–cytokine receptor interaction, and inflammatory pathways. **F**. Heatmap of PI3K-Akt pathway-associated genes reveals significant changes in key genes, such as *SGK1* and *SGK3*, across treatment groups, with red stars indicating genes that were experimentally validated and showed consistent changes with the transcriptomic data. **G**. qPCR validation of PI3K-Akt pathway-related gene expression in BEAS-2B cells treated with bacterial strains (*Cs*, *Ra*, and *Rd*) *vs.* NC. Data are presented as mean ± SD from three independent experiments (*, *P* < 0.05; **, *P* < 0.01; ***, *P* < 0.001; NS, not significant). *Cs*, *Corynebacterium striatum*; *Ra*, *Rothia aeria*; *Rd*, *Rothia dentocariosa*; NC, negative control; PCA, principal component analysis; PC, principal component; DEG, differentially expressed gene; CCK-8, Cell Counting Kit-8; SD, standard deviation; FC, fold change; KEGG, Kyoto Encyclopedia of Genes and Genomes; qPCR, quantitative polymerase chain reaction.

We used LEfSe analysis to investigate the pathogenic potential of three species identified as enriched in the pneumonia group: *Corynebacterium striatum* (*Cs*), *Rothia aeria* (*Ra*), and *Rothia dentocariosa* (*Rd*). Specifically, we evaluated their effects on the viability and gene expression of lung epithelial cells (BEAS-2B). These strains, which have previously been linked to respiratory infections [8,19], were assessed for their ability to induce epithelial BEAS-2B cell damage *in vitro*. Cell Counting Kit-8 (CCK-8) assay revealed that, within 12 h of exposure, all three bacterial strains significantly inhibited cell proliferation to a greater extent in BEAS-2B cells than in the unexposed negative control (NC) cells (**Figure 7**A). Principal component analysis (PCA) of transcriptomic data demonstrated a distinct clustering of the bacterial treatment groups, indicating consistent and reproducible effects on gene expression (Figure 7B). Venn diagram analysis identified 842 differentially expressed genes (DEGs) shared across all three strains, as well as some strain-specific DEGs (Figure 7C). Volcano plots highlighted the extensively upregulated and downregulated DEGs in BEAS-2B cells in response to bacterial exposure (Figure 7D).

The Kyoto Encyclopedia of Genes and Genomes (KEGG) pathway enrichment analysis of the DEGs revealed significant activation of inflammatory pathways, including the PI3K-Akt signaling pathway, cytokine–cytokine receptor interaction, and pathways involved in cancer (Figure 7E). The PI3K-Akt signaling pathway, which regulates inflammation and cell proliferation, was notably enriched, and previous studies have shown that inhibiting this pathway can alleviate pneumonia symptoms [20]. Heatmap analysis revealed further significant changes in key PI3K-Akt-associated genes, such as *SGK1* and *SGK3*, which are critical for cell survival and proliferation (Figure 7F).

We validated these findings by performing real-time reverse-transcription quantitative polymerase chain reaction (RT-qPCR) on 15 PI3K-Akt-related genes in BEAS-2B cells treated with *Cs*, *Ra*, and *Rd*. Compared to NC, the treated cells showed significant dysregulation of several genes: *IRS1*, *ITGA1*, *COL4A4*, and *ITGB8* were significantly downregulated, while *DDIT4*, *EIF4EB*, and *ITGA5* were markedly upregulated (Figure 7G). These changes suggest that the bacteria modulate focal adhesion-related genes and growth factor-related genes, leading to aberrant activation of the PI3K-Akt pathway. This dysregulation likely impacts the downstream mTOR signaling pathway, disrupting normal cellular processes and ultimately inducing cell death.

## Discussion

Despite significant advancements in neonatal care, the diagnosis and management of neonatal pneumonia remain challenging, largely due to the lack of systematic profiles of the neonatal respiratory microbiome and the difficulty in identifying causative pathogens. Traditional cultivation methods often fail to recover slow-growing, low-abundance, or fastidious microbes, thereby impeding accurate diagnoses. The ability to analyze microbial communities has improved with the introduction of metagenomics methods; however, technical challenges continue to limit the application of these methods to the analysis of the neonatal respiratory microbiota. Specifically, the small sample volumes and low microbial biomass typical of neonate sputum samples, as well as the high levels of host DNA contamination, limited recovery of low-abundance species, and high cost of analysis, all hinder conclusive characterization of the microbial populations inhabiting neonatal lungs. To address these limitations, we combined high-throughput amplicon sequencing with microfluidic single-cell cultivation to enable a more comprehensive characterization of the respiratory microbiome involved in neonatal pneumonia. This enhanced capacity to recover marker species underscored the platform’s potential to improve pathogen isolation, identifying it as a valuable tool for advancing neonatal pneumonia diagnostics and understanding the pathogenic mechanisms involved.

Several factors are known to complicate respiratory microbiome profiling and the diagnosis of neonatal pneumonia. For example, early colonization by pathogenic bacteria, such as *Streptococcus pneumoniae* and *P*. *aeruginosa*, often complicates diagnosis, as the coexistence of these pathogens with commensal microbes creates difficulties when attempting to differentiate between infection and colonization [21,22]. Our revelation of pneumonia-associated shifts in microbial composition in the present comparative study highlights the critical role of microbial diversity in neonatal health, particularly given that neonates, especially preterm infants, have underdeveloped lung defenses and immature respiratory microbiomes [1]. Other factors, such as birth mode, gestational age, and antibiotic exposure, can further influence the neonatal microbiome [23,24], thereby increasing susceptibility to infections.

A particularly challenging aspect of determining the pathogens responsible for infections such as neonatal pneumonia is the detection of low-abundance species, which, despite their low relative abundance, play essential roles in maintaining microbial community stability and function. These species may belong to the core microbiome; therefore, their disruption can exacerbate disease progression. However, obtaining uncontaminated respiratory samples in sufficient quantities for direct metagenomic analysis remains difficult due to the low microbial loads and inherent complexity of neonatal respiratory samples [25]. In the present study, although droplet-based single-cell cultivation improved bacterial recovery, several practical challenges were encountered during the cultivation and sequencing workflow. In particular, the volumes and microbial loads of the neonatal sputum samples were often insufficient for direct metagenomic sequencing. The inherently low microbial abundance in these samples also posed challenges for single-cell encapsulation, as reduced cell density can limit interspecies interactions and increase environmental stress within droplets, potentially compromising bacterial viability. We mitigated these issues by introducing a brief pre-enrichment step (2–3 h) prior to droplet encapsulation, and found a moderately increased microbial abundance and an enhanced metabolic activity of certain strains without any significant alteration of the native community structure. This adjustment improved our cultivation success and enabled downstream genomic analyses, ultimately contributing to a more comprehensive and functionally informative microbial dataset. Thus, the integration of high-throughput sequencing with droplet-based cultivation enabled us to address some of the key diagnostic limitations of conventional methods, thereby enhancing the detection of low-abundance species and improving pathogen identification. This combined approach offers a foundation for more precise diagnostics and targeted antimicrobial treatment of neonatal pneumonia.

Our high-throughput sequencing revealed distinct microbial profiles between neonates with and without pneumonia and revealed key microbial shifts linked to respiratory infections. In the pneumonia cases, pathogenic taxa such as *Streptococcus* and *Rothia*, both previously associated with respiratory diseases [26,27], showed significant increases. The β-diversity analysis also demonstrated marked differences in microbial composition between the pneumonia and non-pneumonia groups, indicating that although the overall microbial diversity remained stable, the structure of the microbial communities underwent significant alteration. The compositional changes also showed further correlations with clinical indicators, including postnatal age, gestational age, and hematological markers (*e.g.*, Neu). These findings underscore the potential for using microbiome profiling as a diagnostic tool in neonatal pneumonia, with microbial shifts serving as early indicators of disease severity.

Importantly, our findings showed that low-abundance species contribute significantly to community diversity, but because they are challenging to isolate using traditional methods, they have often been overlooked in metagenomic studies due to difficulties in obtaining their complete genomes for functional analysis. Here, we have shown that advanced techniques, such as high-throughput sequencing and novel cultivation approaches, are crucial to address these limitations and enable more comprehensive and personalized neonatal care. In particular, our droplet-based single-cell cultivation method significantly enhanced microbial recovery and successfully isolated both fastidious and low-abundance species that are often undetectable using traditional methods. This technique allowed us to isolate key pathogens, such as *A*. *baumannii* and *K*. *quasipneumoniae* spp. — ESKAPE pathogens critical to infection control due to their multidrug resistance [28] — as well as other opportunistic species, such as *S*. *pneumoniae* [29] and *C*. *striatum* [18]. The ability to isolate these clinically relevant species is crucial for guiding precise antimicrobial therapies, monitoring antimicrobial susceptibility trends for epidemiologic studies, and reducing the unnecessary use of broad-spectrum antibiotics.

The recovery of low-abundance species, classified based on the original high-throughput sequencing data, also demonstrated the sensitivity of this cultivation method. Our protocol involved the incubation of droplets for seven days to allow the recovery of rare and slow-growing species, and yet rapid bacterial growth was observed in as early as 12 h (Figure 1C), indicating that additional optimization of cultivation time could further shorten diagnostic workflows. This approach also enabled the construction of a more comprehensive microbial profile, which will not only improve diagnostic accuracy but also create a valuable strain collection for future strain-level genome sequencing, pathogenic mechanism studies, and functional analyses, ultimately contributing to the development of novel treatments for neonatal pneumonia. In this study, we were able to leverage this strain collection and perform WGS on 167 representative isolates. This resulted in the identification of 80 distinct species as well as genome-level characteristics, including taxonomic identity and annotated ARGs (Figure 6), which provided strain-specific insights into pathogenicity and drug resistance potential. These genome-resolved data expand the current microbial reference bank and lay the foundation for downstream comparative genomics and biomarker discovery.

Our exploration of the molecular mechanisms of bacterial infection using co-culture experiments with bacterial strains and BEAS-2B cells, followed by transcriptomic analysis, revealed significant upregulation of inflammation-related and immune-related genes, such as *CCL20*, *CSF2*, and *GFRA2*, indicating that bacterial infection triggered an inflammatory response. KEGG pathway analysis revealed enrichment of the PI3K-Akt signaling pathway, which is known to regulate protein synthesis, cell proliferation, and survival. Thus, dysregulation of this pathway, including downregulation of focal adhesion genes such as *COL4A4* and *ITGB8*, may destabilize tissue architecture and suppress airway repair in the neonate lung. A reduction in *PIK3R3* expression may also enhance phosphorylation, leading to PI3K-Akt overactivation and subsequent effects on the downstream mTOR signaling pathway, leading to the disruption of normal protein assembly processes in cells and ultimately to cell death. In neonates with immature immune systems, this overactivation may worsen tissue damage and bacterial infection, implicating the PI3K-Akt pathway in neonatal pneumonia progression.

While our study provides valuable insights into the respiratory microbiome associated with neonatal pneumonia, several limitations should be acknowledged. First, the cross-sectional design of this study restricts our ability to capture dynamic changes in the respiratory microbiome over time. Longitudinal studies are needed to better understand how the microbiome evolves during pneumonia progression, treatment, and recovery. Second, the relatively small sample size may reduce the strength of the conclusions and limit how well the results apply to broader neonatal populations. Because microbial communities are influenced by geographic location, environmental exposures, and healthcare practices, validation of our findings in larger and more diverse cohorts is essential. Third, our analysis focused solely on bacterial communities while excluding viruses and fungi, despite the increasing recognition of these organisms as important contributors to respiratory infections. Future studies should incorporate multi-kingdom microbial profiling to provide a more comprehensive view. Lastly, while we identified correlations between microbial composition and clinical indicators, these associations do not establish causality. Mechanistic studies are required to elucidate the specific roles of key microbial taxa in driving the inflammation, epithelial damage, and disease progression associated with neonatal pneumonia.

In conclusion, this study presents a practical and innovative approach that combines high-throughput sequencing with droplet-based single-cell cultivation to enhance the detection and characterization of the respiratory microbiome in neonates with pneumonia. By identifying key pathogenic species and facilitating the recovery of diverse low-abundance microbes, this method significantly improves our understanding of the baseline neonatal respiratory microbiota and its structural shifts occurring during infection. Host–bacteria interaction experiments provide additional valuable insights into potential pathogenic mechanisms, offering a foundation for future research into targeted therapeutic interventions and a deeper understanding of neonatal pneumonia pathogenesis. Importantly, this combined approach is not limited to explorations of neonatal pneumonia, as it can also be applied to other clinical disorders, such as bloodstream infections and infections at sterile sites, which may also be limited in terms of sample volumes and microbial abundances. This makes our protocol a versatile tool for improving diagnosis and treatment strategies across a broad range of infectious diseases. Overall, the findings reported here provide a meaningful step forward in both microbiome research and clinical diagnostics and have the potential to transform current approaches to infectious diseases in vulnerable populations.

## Materials and methods

### Study cohort and sample collection

A total of 140 neonates were enrolled in this study, and 120 of those neonates provided samples for sequencing. The 120 sampled neonates included 71 cases with neonatal pneumonia and 49 non-pneumonia controls. All clinical diagnoses were independently confirmed by two experienced pediatricians, with a third senior pediatrician providing the final diagnosis, after review and discussion, in cases of disagreement. Sequencing was performed on the original sputum samples and pooled bacterial communities from traditional and droplet-based cultivation. Additionally, due to the limited sample volumes, 20 neonatal samples were used specifically for bacterial isolation and identification using droplet-based cultivation without direct sequencing.

The inclusion criteria for neonatal pneumonia were the presence of clinical symptoms, such as respiratory distress, coughing, pulmonary rales, and fever, confirmed by X-ray examination. Participants also needed to have complete clinical data available. Exclusion criteria included other pulmonary diseases, significant organ dysfunction, prior antimicrobial therapy before admission, congenital diseases, and hereditary metabolic disorders.

### High-throughput picoliter droplet-based single-cell cultivation

Microfluidic devices were fabricated using polydimethylsiloxane (PDMS) via rapid prototyping soft lithography following established protocols [30]. The flow-focusing droplet generation chip design was adapted from a previous study [31] and featured reduced channel widths and a channel height of 40 µm, optimized for producing droplets with a volume of 30 pl at a throughput of up to 8000 droplets per second.

Sputum samples were filtered through a 20-µm filter and suspended in Columbia medium. Bacterial concentrations were estimated using a customized cell counting chip [32] and adjusted to achieve a Poisson distribution with λ < 0.3, thereby minimizing the probability of encapsulating multiple bacterial cells within a single droplet. The bacterial suspension was introduced into the dispersed phase channel at a flow rate of 900 µl/h, while EvaGreen Droplet Generation Oil (Catalog No. 186-4035, Bio-Rad, Hercules, CA) was introduced into the continuous oil phase channel at 1800 µl/h. We used dual-channel syringe pumps (Catalog No. 70-402, Harvard Apparatus, Holliston, MA) for simultaneous droplet generation with two microfluidic devices, producing approximately 1 × 10^8^ droplets with a total volume of 3 ml in less than 100 min, thereby ensuring that over 2 × 10^7^ droplets contained individual bacterial cells. The droplets were collected in Eppendorf tubes and incubated at 37°C for seven days.

Following incubation, we confirmed bacterial growth in the droplets using inverted microscopy (ECLIPSE Ti, Nikon, Tokyo, Japan). Droplets were demulsified by adding an equal volume of 1H,1H,2H,2H-perfluoro-1-octanol (PFO; Catalog No. 647-42-7, Aladdin Bio-Chem Technology, Shanghai, China), followed by vortexing and centrifugation. The layer containing oil and PFO was then carefully removed, and the aqueous phase was collected for DNA extraction. In parallel with the droplet-based cultivation, equal inocula were plated on Columbia agar plates and cultured at 37°C for 12 h. Bacterial colonies from both the agar plates and the droplet-based cultures were then pooled for high-throughput sequencing.

### Bacterial isolation and identification

We subjected 20 neonatal sputum samples to droplet-based cultivation. The droplets were incubated at 37°C for seven days, followed by demulsification and dilution for plating on agar plates. The plates were then incubated at 37°C until visible bacterial colonies formed. Colonies displaying distinct morphologies were selected and transferred to liquid cultures for further growth and identification.

### DNA preparation and sequencing

Genomic DNA was extracted from individual colonies for taxonomic identification of the cultivated bacterial strains. The 16S rRNA gene was amplified using universal primers 27F (5′-AGAGTTTGATCCTGGCTCAG-3′) and 1492R (5′-GGTTACCTTGTTACGACTT-3′), purified, and sequenced on an ABI 3730 automated DNA sequencer.

Microbial genomic DNA was extracted from the original sputum samples, from bacterial communities grown on agar plates (agar pool), and from bacteria grown using droplet-based cultivation (droplet pool). All DNA extractions were conducted using the MagAttract PowerSoil Pro DNA Kit (Catalog No. 47119, QIAGEN, Hilden, Germany) according to the manufacturer’s protocol. The quality and concentration of the extracted DNA were evaluated using 1.0% agarose gel electrophoresis and a NanoDrop2000 spectrophotometer (Catalog No. ND-2000, Thermo Fisher Scientific, Waltham, MA). The hypervariable V3–V4 region of the bacterial 16S rRNA gene was amplified with the primer pairs 338F (5′-ACTCCTACGGGAGGCAGCAG-3′) and 806R (5′-GGACTACHVGGGTWTCTAAT-3′). Sequencing was carried out on the Illumina MiSeq platform (Majorbio Bio-pharm Technology, Shanghai, China).

### Host–pathogen interaction assays

The human BEAS-2B bronchial epithelial cell line (American Type Culture Collection) was used for host–pathogen interaction assays. The bacterial species *Cs*, *Ra*, and *Rd*, identified as significantly enriched in the pneumonia group via LEfSe analysis, were selected for these assays. BEAS-2B cells were seeded in 96-well plates at a density of 2 × 10^4^ cells per well and incubated at 37°C in 5% CO_2_ for 4 h. Bacteria were added at a multiplicity of infection (MOI) of 10 (2 × 10^5^ cells per well) using phosphate-buffered saline (PBS) as a blank control. Following the manufacturer’s instructions, cell viability was evaluated at 0, 6, and 12 h using the CCK-8 (Catalog No. CK04, Dojindo, Kumamoto, Japan). Absorbance at 450 nm was measured as an indicator of metabolic activity and cell viability. Following incubation, the cells were washed three times with PBS, and total RNA was extracted using the Total RNA Extraction Reagent (Catalog No. R401-01, Vazyme, Nanjing, China). RNA quality was verified using a NanoDrop spectrophotometer, and the samples were submitted to Majorbio Bio-pharm Technology for RNA sequencing (RNA-seq).

### RNA extraction and RT-qPCR analysis

Cell samples were collected and washed three times with PBS. Total RNA was extracted using TRIzol reagent and reverse-transcribed into complementary DNA (cDNA) using the HiScript III All-in-one RT SuperMix Perfect for qPCR (Catalog No. R333-01, Vazyme). Quantitative PCR (qPCR) was performed with the Taq Pro Universal SYBR qPCR Master Mix (Catalog No. Q712-02, Vazyme) on a Quantagene q225MX qPCR system (Kubo Technology, Beijing, China). Data were presented as fold change using the 2^−ΔΔCt^ method. All designed primers were purchased from Shanghai Beyotime Biotech Inc., China.

### Bioinformatic and statistical analyses

For species identification, full-length 16S rRNA gene sequences were assembled using DNASTAR software and compared against the EzBioCloud database (http://www.ezbiocloud.net/). For high-throughput 16S rRNA sequencing, paired-end reads were merged using Fast Length Adjustment of SHort reads (FLASH) [33], and quality control was performed with Quantitative Insights Into Microbial Ecology 2 (QIIME 2). Reads were clustered into operational taxonomic units (OTUs) at 97% similarity using USEARCH. Representative OTU sequences were annotated using the Ribosomal Database Project (RDP) classifier. Diversity analysis, LEfSe analysis, and functional prediction were conducted via the Majorbio Cloud platform (https://www.majorbio.com).

For RNA-seq data, the raw paired-end reads were trimmed and quality controlled using fastp with default parameters. Clean reads were aligned to the reference genome using the orientation mode in Hierarchical Indexing for Spliced Alignment of Transcripts 2 (HISAT2) software. The mapped reads of each sample were assembled using StringTie in a reference-based approach. DEGs between samples were identified by calculating transcript expression levels using the transcripts per million (TPM) method. Functional enrichment analyses, including Gene Ontology (GO) and KEGG pathway analyses, were performed to identify significantly enriched DEGs. Enrichment was considered significant at a Bonferroni-corrected *P* value < 0.05 compared with the whole transcriptome background. GO and KEGG pathway enrichment analyses were carried out using GOATOOLS and Python scipy software, respectively.

Statistical analyses were performed using Statistical Product and Service Solutions (SPSS; v26.0, Chicago). Data are presented as mean ± standard deviation. Statistical significance was determined using Chi-squared tests and unpaired *t*-tests, with a *P* value < 0.05 considered significant.

## Ethical statement

The study was approved by the Ethics Committee of Beijing Friendship Hospital, Capital Medical University, China (Approval No. L-2021-096). Written informed consent was obtained from both parents of each neonate participant before enrollment.

## Supplementary Material

qzaf047_Supplementary_Data

## Data Availability

Raw sequence data obtained in this study have been deposited in the Genome Sequence Archive [34] at the National Genomics Data Center (NGDC), China National Center for Bioinformation (CNCB) (GSA: CRA024783) that are publicly accessible at https://ngdc.cncb.ac.cn/gsa.

## CRediT author statement

**Zerui Wang:** Conceptualization, Methodology, Software, Validation, Formal analysis, Investigation, Data curation, Writing – original draft, Writing – review & editing, Visualization. **Xin Cheng:** Investigation, Data curation, Writing – review & editing. **Yibin Xu:** Investigation, Data curation, Writing – review & editing. **Zhiyi Wang:** Investigation. **Liyan Ma:** Resources, Writing – review & editing. **Caiming Li:** Writing – review & editing, Visualization. **Shize Jiang:** Investigation. **Yuchen Li:** Investigation. **Shuilong Guo:** Resources, Writing – review & editing. **Wenbin Du:** Supervision, Fund acquisition, Conceptualization, Formal analysis, Writing – review & editing, Visualization. All authors read and approved the final manuscript.
