# Supplementary material for: Unveiling Neonatal Pneumonia Microbiome by High-throughput Sequencing and Droplet Culturomics
Source: Genomics Proteomics Bioinformatics. 2025 May 29;23(6):qzaf047. doi: 10.1093/gpbjnl/qzaf047 (PMC12721867; doi:10.1093/gpbjnl/qzaf047)
Supplement: qzaf047_Supplementary_Data [file qzaf047_supplementary_data.zip › GPB-D-25-00044 052625_supplement.pdf]

Supplementary material

File S1 Supplementary methods

Figure S1 α-diversity analysis between neonatal pneumonia and non-pneumonia groups

A.–D. 16S rRNA sequencing-based diversity comparison between neonatal pneumonia and non-pneumonia groups based on α-diversity indices, including Shannon (P = 0.100) (A), Sobs (P = 0.066) (B), Simpson (P = 0.690) (C), and Coverage (P = 0.179) (D). P values indicate the differences between neonatal pneumonia and non-pneumonia groups for each index. **E.** Bray-Curtis dissimilarity boxplots showing within-group (neonatal pneumonia and non-pneumonia) and between-group distances at the genus level (P = 0.006).

Figure S2 Relative abundance of genus-level differential species in 120 neonatal sputum samples

This figure shows the relative proportions of genera that differ significantly between neonatal pneumonia and non-pneumonia groups, as determined by LEfSe analysis. Genera such as *Streptococcus*, *Rothia*, and *Pseudomonas* are more abundant in the pneumonia group, while *Achromobacter*, *Acinetobacter*, *Pedobacter*, and others are more enriched in the non-pneumonia group. (*, $P < 0.05$; **, $P < 0.01$; ***, $P < 0.001$).

Figure S3 Network analysis of differential bacterial genera for pneumonia and non-pneumonia neonates

A. and **B.** This network analysis shows significant interactions among bacterial genera in neonatal pneumonia (A) and non-pneumonia (B) groups at the genus level. The size of each node reflects the abundance of the corresponding genus, and node color corresponds to its phylum classification. Red edges indicate positive correlations, while green edges represent negative correlations. The thickness of each edge is proportional to the strength of the correlation. Only associations with a Spearman correlation coefficient greater than 0.5 (absolute value) and a P value less than 0.05 were considered significant.

Figure S4 Functional predictions of neonatal respiratory microbiome using PICRUSt2

A. Predicted KEGG pathways with significant differences between neonatal pneumonia and non-pneumonia groups, showing enriched functions in purine metabolism, ribosome formation, and amino acid biosynthesis in pneumonia samples. **B.** Predicted MetaCyc pathways, highlighting differences such as L-isoleucine biosynthesis III and aerobic respiration in non-pneumonia samples, and branched-chain amino acid biosynthesis and peptidoglycan maturation in pneumonia samples. **C.** Enzyme-level predictions showing higher levels of histidine kinase, DNA helicase, and DNA-directed DNA polymerase in pneumonia samples, and 3-oxoacyl-[acyl-carrier-protein] reductase and peptidylprolyl isomerase in non-pneumonia samples. Differences are shown with 95% confidence intervals (*, $P < 0.05$; **, $P < 0.01$; Welch's *t*-test, FDR adjusted). FDR, false discovery rate; PICRUSt2, Phylogenetic Investigation of Communities by Reconstruction of Unobserved States 2; MetaCyc, Metabolic Pathways From all Domains of Life.

Table S1 Culture rates of different bacteria of LEfSe analysis at the species level between droplets and agar plates

Table S2 Bacterial strains isolated from neonatal sputum with droplet-based cultivation

Table S3 Assessment of genome completeness of isolates based on BUSCO analysis

File S1 Supplementary methods

Appendix result 1: predicted microbial metabolisms in neonatal pneumonia and non-pneumonia groups

Microbial community functions were predicted using the Phylogenetic Investigation of Communities by Reconstruction of Unobserved States 2 (PICRUSt2) platform. Across the third-level Kyoto Encyclopedia of Genes and Genomes (KEGG) pathways, functions related to purine metabolism, ribosome formation, amino acid biosynthesis, and secondary metabolite biosynthesis were more abundant in the neonatal pneumonia group, while pathways associated with the two-component system and microbial metabolism were enriched in the non-pneumonia group (Figure S4A). The elevated purine metabolism and ribosome formation in the pneumonia group may reflect increased bacterial growth and virulence, contributing to more severe clinical symptoms such as inflammation and respiratory distress.

Further annotation using Metabolic Pathways From all Domains of Life (MetaCyc) metabolic pathway database revealed the pathways involved in the non-oxidative branch of the pentose phosphate pathway, branched-chain amino acid biosynthesis, and peptidoglycan maturation were more prevalent in the neonatal pneumonia group. Conversely, pathways for L-isoleucine biosynthesis, fatty acid salvage, and aerobic respiration I (cytochrome C) were more prominent in the non-pneumonia group (Figure S4B). These findings suggest that pneumonia-associated bacteria rely on metabolic adaptations to thrive in the host environment, promoting disease progression.

Enzymatic analysis revealed that histidine kinase, DNA helicase, and DNA-directed DNA polymerase were more abundant in the pneumonia group, indicating active bacterial replication and survival mechanisms, potentially contributing to the persistence of infection. In contrast, 3-oxoacyl-[acyl-carrier-protein] reductase and peptidylprolyl isomerase were enriched in the non-pneumonia group (Figure S4C), aligning with a more stable, commensal microbiome that may help maintain respiratory health. These metabolic differences likely correlate with the severity and progression of clinical symptoms in neonatal pneumonia.

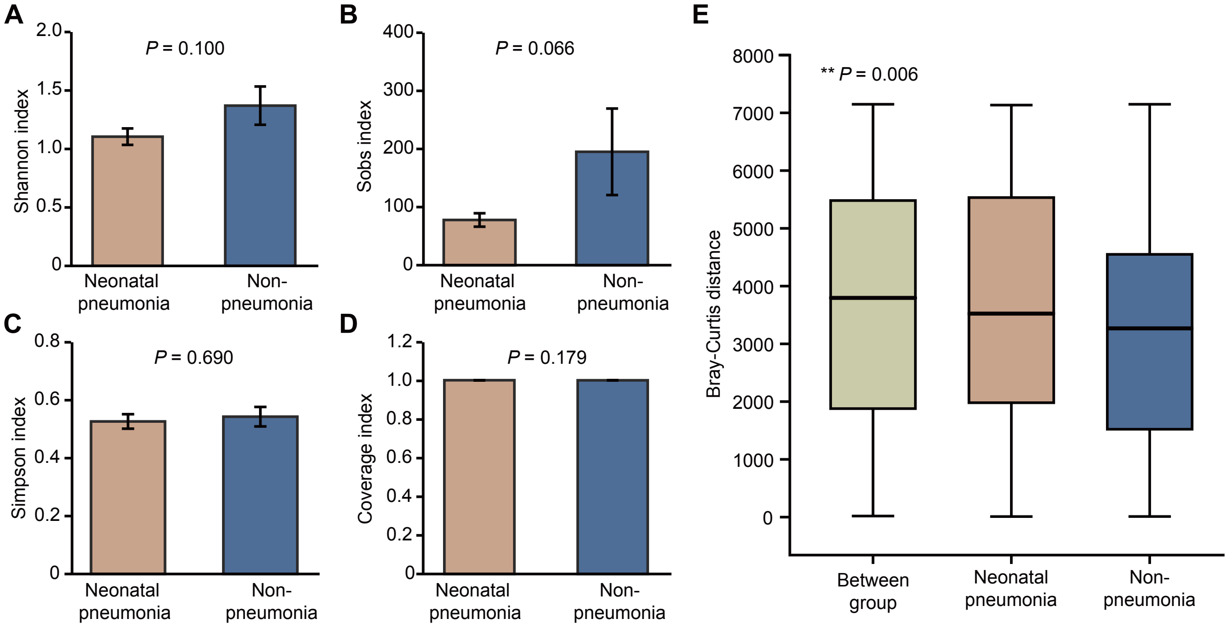
A
Shannon index
P = 0.100
Neonatal pneumonia
Non-pneumonia
B
Sobs index
P = 0.066
C
Simpson index
P = 0.690
D
Coverage index
P = 0.179
E
Bray-Curtis distance
** P = 0.006
Between group
Neonatal pneumonia
Non-pneumonia

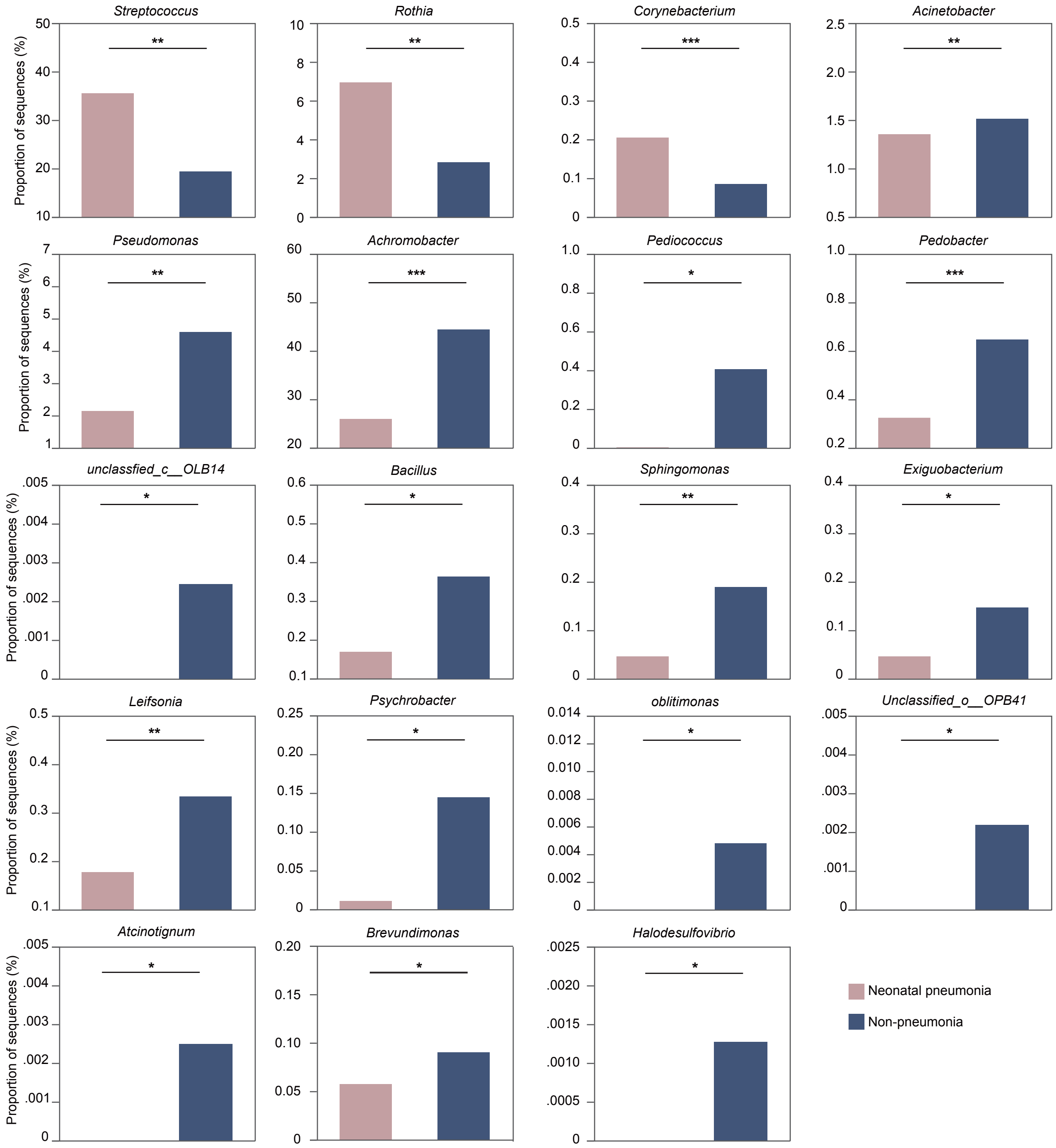

Streptococcus
Rothia
Corynebacterium
Acinetobacter
Pseudomonas
Achromobacter
Pediococcus
Pedobacter
unclassfied_c__OLB14
Bacillus
Sphingomonas
Exiguobacterium
Leifsonia
Psychrobacter
oblitimonas
Unclassified_o__OPB41
Atcinotignum
Brevundimonas
Halodesulfovibrio
Proportion of sequences (%)
Neonatal pneumonia
Non-pneumonia

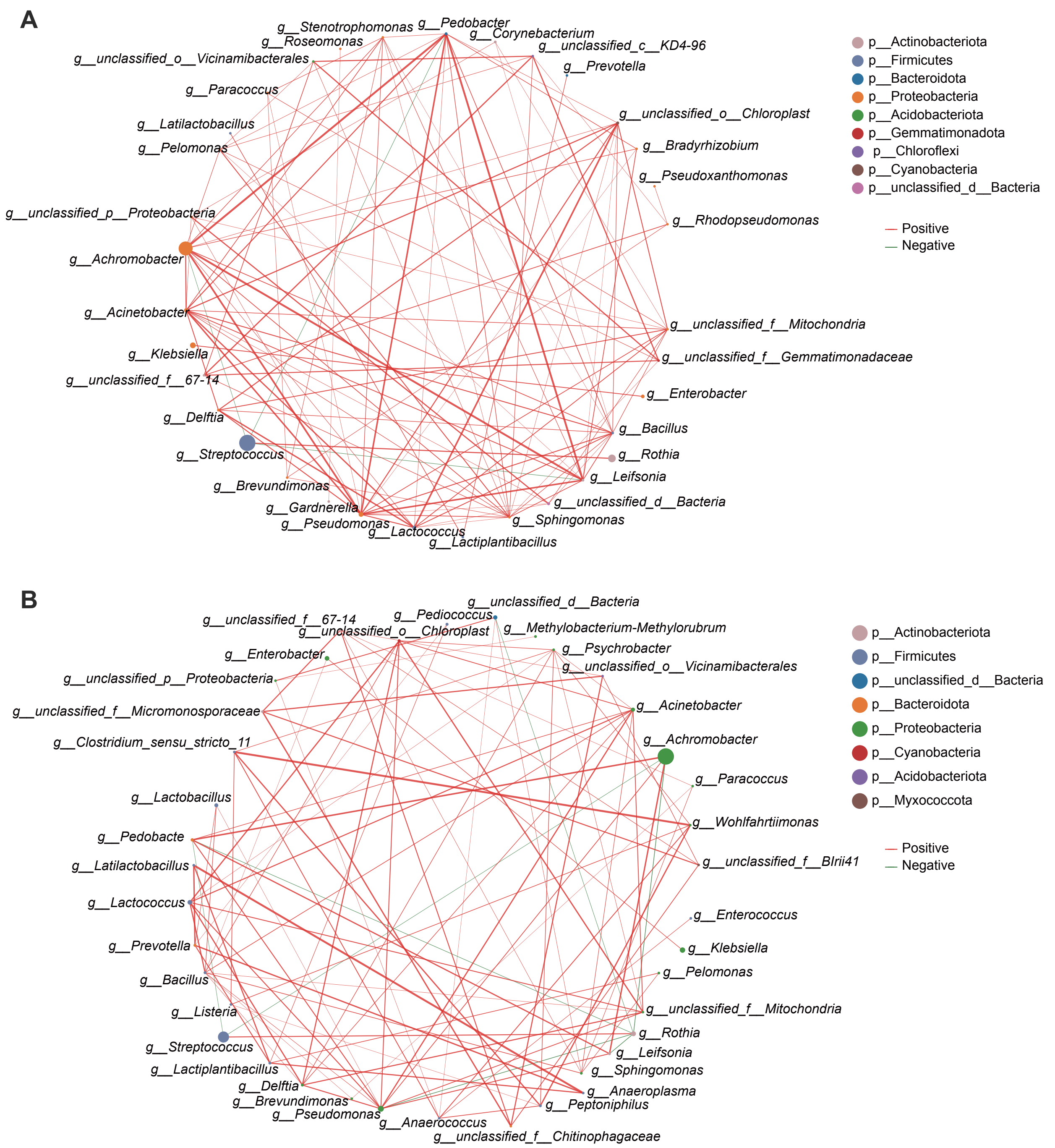
A
g__Stenotrophomonas
g__Pedobacter
g__Roseomonas
g__Corynebacterium
g__unclassified_c__KD4-96
g__unclassified_o__Vicinamibacterales
g__Prevotella
g__Paracoccus
g__unclassified_o__Chloroplast
g__Latilactobacillus
g__Bradyrhizobium
g__Pelomonas
g__Pseudoxanthomonas
g__unclassified_p__Proteobacteria
g__Rhodopseudomonas
g__Achromobacter
g__Acinetobacter
g__unclassified_f__Mitochondria
g__Klebsiella
g__unclassified_f__Gemmatimonadaceae
g__unclassified_f__67-14
g__Enterobacter
g__Delftia
g__Bacillus
g__Streptococcus
g__Rothia
g__Leifsonia
g__Brevundimonas
g__unclassified_d__Bacteria
g__Gardnerella
g__Sphingomonas
g__Pseudomonas
g__Lactococcus
g__Lactiplantibacillus
p__Actinobacteriota
p__Firmicutes
p__Bacteroidota
p__Proteobacteria
p__Acidobacteriota
p__Gemmatimonadota
p__Chloroflexi
p__Cyanobacteria
p__unclassified_d__Bacteria
Positive
Negative
B
g__unclassified_d__Bacteria
g__unclassified_f__67-14
g__Pediococcus
g__unclassified_o__Chloroplast
g__Methylobacterium-Methylorubrum
g__Psychrobacter
g__Enterobacter
g__unclassified_o__Vicinamibacterales
g__unclassified_p__Proteobacteria
g__Acinetobacter
g__unclassified_f__Micromonosporaceae
g__Achromobacter
g__Clostridium_sensu_stricto_11
g__Paracoccus
g__Lactobacillus
g__Wohlfahrtiimonas
g__Pedobacte
g__unclassified_f__Blrii41
g__Latilactobacillus
g__Lactococcus
g__Enterococcus
g__Prevotella
g__Klebsiella
g__Bacillus
g__Pelomonas
g__Listeria
g__unclassified_f__Mitochondria
g__Rothia
g__Streptococcus
g__Leifsonia
g__Lactiplantibacillus
g__Sphingomonas
g__Delftia
g__Anaeroplasma
g__Brevundimonas
g__Peptoniphilus
g__Pseudomonas
g__Anaerococcus
g__unclassified_f__Chitinophagaceae
p__Actinobacteriota
p__Firmicutes
p__unclassified_d__Bacteria
p__Bacteroidota
p__Proteobacteria
p__Cyanobacteria
p__Acidobacteriota
p__Myxococcota
Positive
Negative

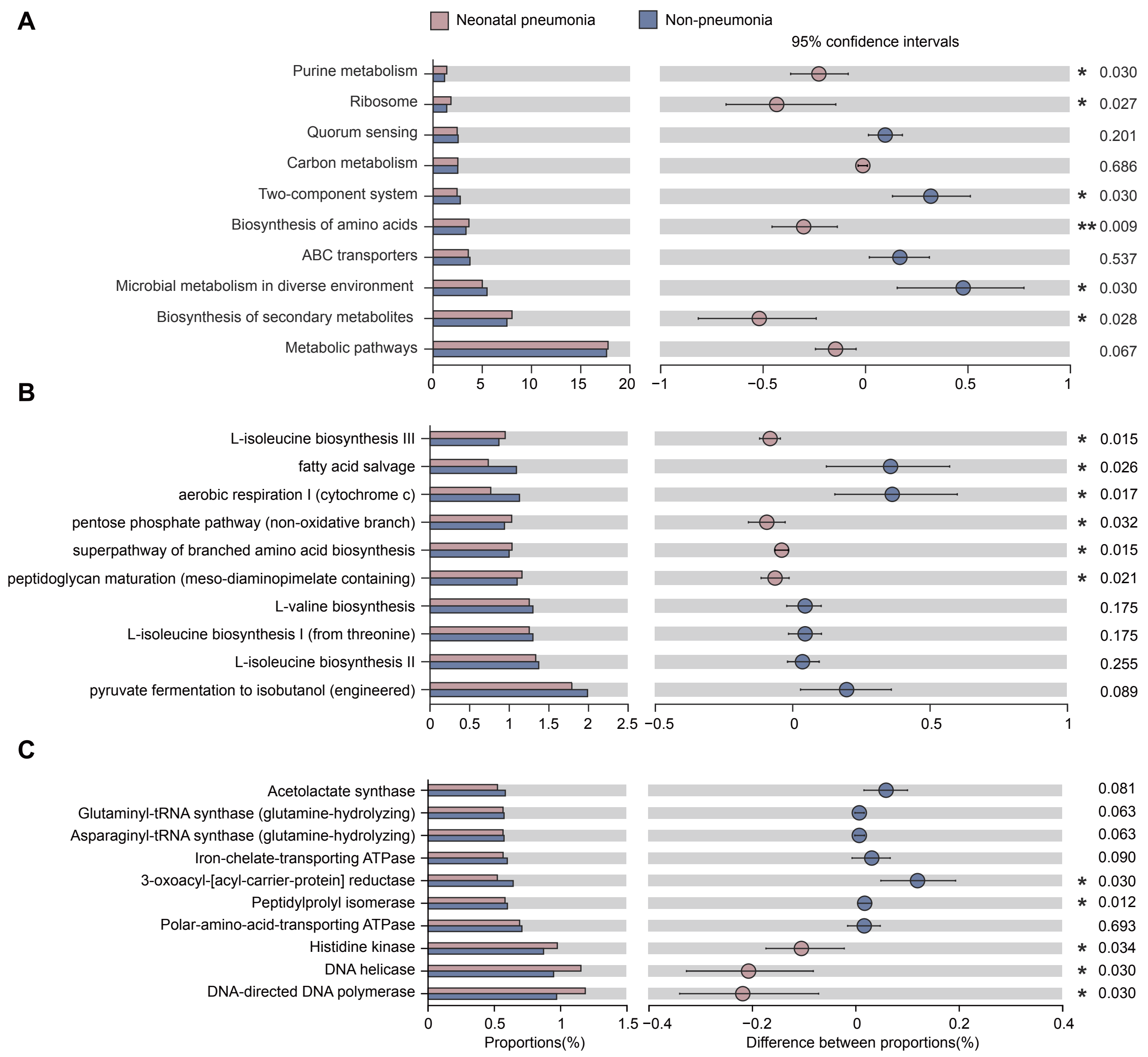

A
Neonatal pneumonia
Non-pneumonia
95% confidence intervals
Purine metabolism * 0.030
Ribosome * 0.027
Quorum sensing 0.201
Carbon metabolism 0.686
Two-component system * 0.030
Biosynthesis of amino acids ** 0.009
ABC transporters 0.537
Microbial metabolism in diverse environment * 0.030
Biosynthesis of secondary metabolites * 0.028
Metabolic pathways 0.067
0 5 10 15 20
−1 −0.5 0 0.5 1
B
L-isoleucine biosynthesis III * 0.015
fatty acid salvage * 0.026
aerobic respiration I (cytochrome c) * 0.017
pentose phosphate pathway (non-oxidative branch) * 0.032
superpathway of branched amino acid biosynthesis * 0.015
peptidoglycan maturation (meso-diaminopimelate containing) * 0.021
L-valine biosynthesis 0.175
L-isoleucine biosynthesis I (from threonine) 0.175
L-isoleucine biosynthesis II 0.255
pyruvate fermentation to isobutanol (engineered) 0.089
0 0.5 1 1.5 2 2.5
−0.5 0 0.5 1
C
Acetolactate synthase 0.081
Glutaminyl-tRNA synthase (glutamine-hydrolyzing) 0.063
Asparaginyl-tRNA synthase (glutamine-hydrolyzing) 0.063
Iron-chelate-transporting ATPase 0.090
3-oxoacyl-[acyl-carrier-protein] reductase * 0.030
Peptidylprolyl isomerase * 0.012
Polar-amino-acid-transporting ATPase 0.693
Histidine kinase * 0.034
DNA helicase * 0.030
DNA-directed DNA polymerase * 0.030
0 0.5 1 1.5
−0.4 −0.2 0 0.2 0.4
Proportions(%)
Difference between proportions(%)

File S1 Supplementary methods

Appendix result 1: predicted microbial metabolisms in neonatal pneumonia and non-pneumonia groups

Microbial community functions were predicted using the Phylogenetic Investigation of Communities by Reconstruction of Unobserved States 2 (PICRUSt2) platform. Across the third-level Kyoto Encyclopedia of Genes and Genomes (KEGG) pathways, functions related to purine metabolism, ribosome formation, amino acid biosynthesis, and secondary metabolite biosynthesis were more abundant in the neonatal pneumonia group, while pathways associated with the two-component system and microbial metabolism were enriched in the non-pneumonia group (Figure S4A). The elevated purine metabolism and ribosome formation in the pneumonia group may reflect increased bacterial growth and virulence, contributing to more severe clinical symptoms such as inflammation and respiratory distress.

Further annotation using Metabolic Pathways From all Domains of Life (MetaCyc) metabolic pathway database revealed the pathways involved in the non-oxidative branch of the pentose phosphate pathway, branched-chain amino acid biosynthesis, and peptidoglycan maturation were more prevalent in the neonatal pneumonia group. Conversely, pathways for L-isoleucine biosynthesis, fatty acid salvage, and aerobic respiration I (cytochrome C) were more prominent in the non-pneumonia group (Figure S4B). These findings suggest that pneumonia-associated bacteria rely on metabolic adaptations to thrive in the host environment, promoting disease progression.

Enzymatic analysis revealed that histidine kinase, DNA helicase, and DNA-directed DNA polymerase were more abundant in the pneumonia group, indicating active bacterial replication and survival mechanisms, potentially contributing to the persistence of infection. In contrast, 3-oxoacyl-[acyl-carrier-protein] reductase and peptidylprolyl isomerase were enriched in the non-pneumonia group (Figure S4C), aligning with a more stable, commensal microbiome that may help maintain respiratory health. These metabolic differences likely correlate with the severity and progression of clinical symptoms in neonatal pneumonia.

Table S2 Bacterial strains isolated from neonatal sputum with droplet-based cultivation

Isolate number	Phylogenetic affiliation	Closest cultivated species	Isolates	Percentage of isolates
1	Actinobacteria	*Actinomyces oris*	6	2.01%
2	Actinobacteria	*Rothia endophytica*	5	1.67%
3	Actinobacteria	*Rothia mucilaginosa*	5	1.67%
4	Actinobacteria	*Rothia amarae*	5	1.67%
5	Actinobacteria	*Actinomyces naeslundii*	2	0.67%
6	Actinobacteria	*Kocuria rosea*	2	0.67%
7	Actinobacteria	*Microbacterium testaceum*	2	0.67%
8	Actinobacteria	*Rothia aeria*	2	0.67%
9	Actinobacteria	*Rothia dentocariosa*	2	0.67%
10	Actinobacteria	*Corynebacterium durum*	1	0.33%
11	Actinobacteria	*Corynebacterium striatum*	1	0.33%
12	Actinobacteria	*Curtobacterium luteum*	1	0.33%
13	Actinobacteria	*Microbacterium zeae*	1	0.33%
14	Actinobacteria	*Micrococcus endophyticus*	1	0.33%
15	Actinobacteria	*Micrococcus flavus*	1	0.33%
16	Actinobacteria	*Rhodococcus ruber*	1	0.33%
17	Actinobacteria	*Rothia marina*	1	0.33%
18	Firmicutes	*Staphylococcus argenteus*	16	5.35%
19	Firmicutes	*Streptococcus vestibularis*	15	5.02%
20	Firmicutes	*Staphylococcus epidermidis*	14	4.68%
21	Firmicutes	*Streptococcus salivarius*	12	4.01%
22	Firmicutes	*Streptococcus ilei*	9	3.01%
23	Firmicutes	*Staphylococcus hominis subsp. novobiosepticus*	7	2.34%
24	Firmicutes	*Streptococcus oralis subsp. dentisani*	7	2.34%
25	Firmicutes	*Streptococcus sanguinis*	7	2.34%
26	Firmicutes	*Staphylococcus haemolyticus*	6	2.01%
27	Firmicutes	*Abiotrophia defectiva*	5	1.67%
28	Firmicutes	*Streptococcus gordonii*	5	1.67%
29	Firmicutes	*Streptococcus lactarius*	5	1.67%
30	Firmicutes	*Staphylococcus lugdunensis*	4	1.34%
31	Firmicutes	*Streptococcus downii*	4	1.34%
32	Firmicutes	*Streptococcus mitis*	4	1.34%

33	Firmicutes	*Enterococcus faecalis*	3	1.00%
34	Firmicutes	*Streptococcus parasanguinis*	3	1.00%
35	Firmicutes	*Bacillus altitudinis*	2	0.67%
36	Firmicutes	*Cytobacillus firmus*	2	0.67%
37	Firmicutes	*Lactobacillus paragasseri*	2	0.67%
38	Firmicutes	*Niallia circulans*	2	0.67%
39	Firmicutes	*Priestia aryabhattai*	2	0.67%
40	Firmicutes	*Streptococcus peroris*	2	0.67%
41	Firmicutes	*Streptococcus symci*	2	0.67%
42	Firmicutes	*Streptococcus gwangjuense*	2	0.67%
43	Firmicutes	*Streptococcus infantis*	2	0.67%
44	Firmicutes	*Streptococcus shenyangsis*	2	0.67%
45	Firmicutes	*Streptococcus koreensis*	2	0.67%
46	Firmicutes	*Streptococcus cristatus*	2	0.67%
47	Firmicutes	*Streptococcus australis*	2	0.67%
48	Firmicutes	*Streptococcus panodentis*	2	0.67%
49	Firmicutes	*Streptococcus bouchesdurhonensis*	2	0.67%
50	Firmicutes	*Streptococcus anginosus subsp. anginosus*	2	0.67%
51	Firmicutes	*Bacillus cereus*	1	0.33%
52	Firmicutes	*Bacillus pacificus*	1	0.33%
53	Firmicutes	*Bacillus paralicheniformis*	1	0.33%
54	Firmicutes	*Bacillus wiedmannii*	1	0.33%
55	Firmicutes	*Bacillus safensis subsp. safensis*	1	0.33%
56	Firmicutes	*Bacillus infantis*	1	0.33%
57	Firmicutes	*Cytobacillus pseudoceanisediminis*	1	0.33%
58	Firmicutes	*Fictibacillus barbaricus*	1	0.33%
59	Firmicutes	*Gemella sanguinis*	1	0.33%
60	Firmicutes	*Granulicatella adiacens*	1	0.33%
61	Firmicutes	*Metabacillus idriensis*	1	0.33%
62	Firmicutes	*Neobacillus drentensis*	1	0.33%
63	Firmicutes	*Peribacillus asahii*	1	0.33%
64	Firmicutes	*Priestia megaterium*	1	0.33%
65	Firmicutes	*Rossellomorea marisflavi*	1	0.33%
66	Firmicutes	*Staphylococcus caprae*	1	0.33%
67	Firmicutes	*Staphylococcus capitis subsp. capitis*	1	0.33%
68	Firmicutes	*Streptococcus thermophilus*	1	0.33%

69	Firmicutes	*Streptococcus timonensis*	1	0.33%
70	Firmicutes	*Streptococcus humanilactis*	1	0.33%
71	Firmicutes	*Streptococcus pseudopneumoniae*	1	0.33%
72	Firmicutes	*Streptococcus vulneris*	1	0.33%
73	Firmicutes	*Streptococcus toyakuensis*	1	0.33%
74	Firmicutes	*Streptococcus oralis subsp. tigurinus*	1	0.33%
75	Firmicutes	*Streptococcus chosunense*	1	0.33%
76	Firmicutes	*Streptococcus oralis subsp. oralis*	1	0.33%
77	Firmicutes	*Streptococcus halitosis*	1	0.33%
78	Firmicutes	*Streptococcus pneumoniae*	1	0.33%
79	Firmicutes	*Streptococcus rubneri*	1	0.33%
80	Proteobacteria	*Achromobacter xylosoxidans*	1	0.33%
81	Proteobacteria	*Escherichia fergusonii*	43	14.38%
82	Proteobacteria	*Neisseria perflava*	7	2.34%
83	Proteobacteria	*Klebsiella quasipneumoniae subsp. similipneumoniae*	5	1.67%
84	Proteobacteria	*Pseudomonas aeruginosa*	3	1.00%
85	Proteobacteria	*Acinetobacter schindleri*	2	0.67%
86	Proteobacteria	*Neisseria macacae*	2	0.67%
87	Proteobacteria	*Stenotrophomonas maltophilia*	2	0.67%
88	Proteobacteria	*Acinetobacter baumannii*	1	0.33%
89	Proteobacteria	*Escherichia ruysiae*	1	0.33%
90	Proteobacteria	*Escherichia hominis*	1	0.33%
91	Proteobacteria	*Escherichia whittamii*	1	0.33%
92	Proteobacteria	*Haemophilus parainfluenzae*	1	0.33%
93	Proteobacteria	*Klebsiella quasipneumoniae subsp. quasipneumoniae*	1	0.33%
94	Proteobacteria	*Pelomonas puraquae*	1	0.33%
95	Proteobacteria	*Shigella flexneri*	1	0.33%

Table S3 Assessment of genome completeness of isolates based on BUSCO analysis

Isolate number	Species	BUSCO (100%)
1	*Priestia aryabhattai*	99.2
2	*Micrococcus flavus*	98.4
3	*Rhodococcus ruber*	99.2
4	*Rothia mucilaginosa*	97.6
5	*Streptococcus vestibularis*	99.2
6	*Kocuria rosea*	99.2
7	*Enterococcus faecalis*	98.4
8	*Pseudomonas aeruginosa*	100
9	*Stenotrophomonas maltophilia*	100
10	*Staphylococcus epidermidis*	100
11	*Streptococcus anginosus subsp. anginosus*	98.4
12	*Bacillus wiedmannii*	99.2
13	*Escherichia whittamii*	100
14	*Klebsiella quasipneumoniae subsp. quasipneumoniae*	97.6
15	*Escherichia fergusonii*	100
16	*Escherichia ruysiae*	100
17	*Escherichia hominis*	100
18	*Streptococcus lactarius*	96.8
19	*Staphylococcus argenteus*	100
20	*Klebsiella quasipneumoniae subsp. similipneumoniae*	98.4
21	*Curtobacterium luteum*	96.8
22	*Bacillus pacificus*	100
23	*Niallia circulans*	100
24	*Bacillus safensis subsp. safensis*	100
25	*Metabacillus idriensis*	100
26	*Fictibacillus barbaricus*	100
27	*Cytobacillus pseudoceanisediminis*	100
28	*Peribacillus asahii*	100
29	*Rossellomorea marisflavi*	100
30	*Neobacillus drentensis*	99.2
31	*Micrococcus endophyticus*	99.2
32	*Microbacterium zeae*	97.6
33	*Microbacterium testaceum*	98.4

34	*Acinetobacter baumannii*	98.4
35	*Bacillus paralicheniformis*	100
36	*Acinetobacter schindleri*	100
37	*Actinomyces oris*	100
38	*Streptococcus infantis*	97.6
39	*Streptococcus humanilactis*	99.2
40	*Rothia aeria*	99.2
41	*Streptococcus salivarius*	99.2
42	*Streptococcus bouchesdurhonensis*	99.2
43	*Neisseria perflava*	99.2
44	*Streptococcus toyakuensis*	99.2
45	*Staphylococcus haemolyticus*	100
46	*Streptococcus oralis subsp. dentisani*	99.2
47	*Staphylococcus lugdunensis*	100
48	*Rothia endophytica*	97.6
49	*Streptococcus mitis*	98.4
50	*Bacillus infantis*	98.4
51	*Bacillus altitudinis*	100
52	*Streptococcus symci*	98.4
53	*Cytobacillus firmus*	100
54	*Rothia dentocariosa*	99.2
55	*Rothia amarae*	98.4
56	*Lactobacillus paragasseri*	91.1
57	*Streptococcus ilei*	97.6
58	*Neisseria macacae*	97.6
59	*Streptococcus chosunense*	99.2
60	*Streptococcus parasanguinis*	94.4
61	*Streptococcus oralis subsp. oralis*	99.2
62	*Streptococcus thermophilus*	98.4
63	*Streptococcus bouchesdurhonensis*	99.2
64	*Streptococcus chosunense*	99.2
65	*Streptococcus australis*	99.2
66	*Streptococcus sanguinis*	98.4
67	*Haemophilus parainfluenzae*	99.2
68	*Streptococcus pseudopneumoniae*	98.4
69	*Corynebacterium durum*	99.2

70	*Streptococcus gordonii*	99.2
71	*Rothia marina*	98.4
72	*Staphylococcus caprae*	100
73	*Streptococcus shenyangsis*	99.2
74	*Actinomyces naeslundii*	100
75	*Staphylococcus capitis subsp. capitis*	100
76	*Streptococcus gwangjuense*	99.2
77	*Streptococcus halitosis*	95.1
78	*Streptococcus oralis subsp. tigurinus*	97.6
79	*Bacillus cereus*	100
80	*Streptococcus koreensis*	98.4

Note: BUSCO, Benchmarking Universal Single-Copy Orthologs.

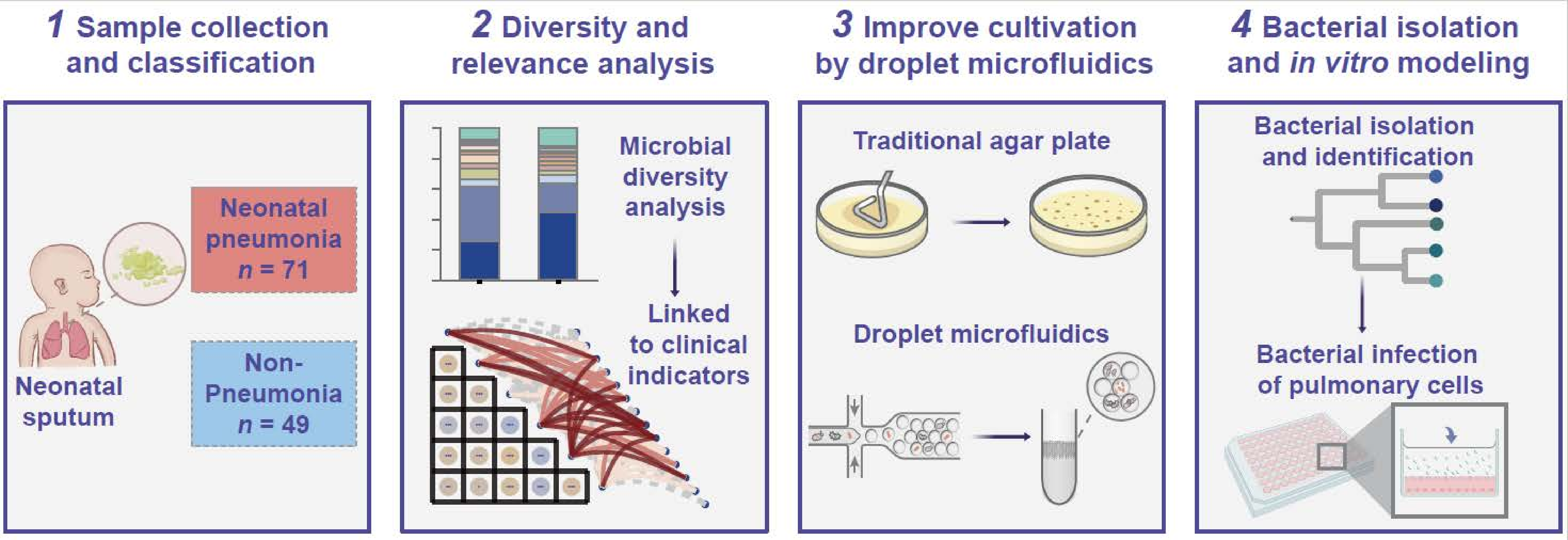
1 Sample collection and classification
Neonatal pneumonia n = 71
Non-Pneumonia n = 49
Neonatal sputum
2 Diversity and relevance analysis
Microbial diversity analysis
Linked to clinical indicators
3 Improve cultivation by droplet microfluidics
Traditional agar plate
Droplet microfluidics
4 Bacterial isolation and in vitro modeling
Bacterial isolation and identification
Bacterial infection of pulmonary cells
